# Clinical Features, Genome Epidemiology, and Antimicrobial Resistance Profiles of *Aeromonas* spp. Causing Human Infections: A Multicenter Prospective Cohort Study

**DOI:** 10.1093/ofid/ofad587

**Published:** 2023-11-16

**Authors:** Aki Sakurai, Masahiro Suzuki, Daisuke Ohkushi, Sohei Harada, Naoto Hosokawa, Kazuhiro Ishikawa, Takayuki Sakurai, Takuma Ishihara, Hiroki Sasazawa, Takeru Yamamoto, Kazumi Takehana, Saho Koyano, Yohei Doi

**Affiliations:** Department of Infectious Diseases, Fujita Health University School of Medicine, Aichi, Japan; Department of Microbiology, Fujita Health University School of Medicine, Aichi, Japan; Department of Microbiology, Fujita Health University School of Medicine, Aichi, Japan; Department of Infectious Diseases, Cancer Institute Hospital, Japanese Foundation for Cancer Research, Tokyo, Japan; Department of Infection Control and Prevention, University of Tokyo Hospital, Tokyo, Japan; Department of Infectious Diseases, Kameda Medical Center, Chiba, Japan; Department of Infectious Diseases, St Luke's International Hospital, Tokyo, Japan; Department of Infectious Diseases, NTT Medical Center, Tokyo, Japan; Innovative and Clinical Research Promotion Center, Gifu University Hospital, Gifu, Japan; Department of Infectious Diseases, Kameda Medical Center, Chiba, Japan; Department of Internal Medicine/Infectious Diseases, Omachi Municipal General Hospital, Nagano, Japan; Department of Infectious Diseases, Kameda Medical Center, Chiba, Japan; Clinical Laboratory, Cancer Institute Hospital, Japanese Foundation for Cancer Research, Tokyo, Japan; Department of Infection Control and Prevention, University of Tokyo Hospital, Tokyo, Japan; Department of Infectious Diseases, Fujita Health University School of Medicine, Aichi, Japan; Department of Microbiology, Fujita Health University School of Medicine, Aichi, Japan; Division of Infectious Diseases, University of Pittsburgh School of Medicine, Pittsburgh, Pennsylvania, USA

**Keywords:** *Aeromonas*, cohort studies, drug resistance, molecular epidemiology, β-Lactam resistance

## Abstract

**Background:**

The genus *Aeromonas* is increasingly implicated in human infections, but knowledge of its clinical characteristics and antimicrobial resistance profiles has been limited owing to its complex taxonomy.

**Methods:**

We conducted a multicenter prospective cohort study of patients with *Aeromonas* infections at hospitals across Japan. Patients were eligible for inclusion if they had an *Aeromonas* spp. strain in a clinical culture and were considered infected at the culture site. Clinical data were collected, and isolates underwent susceptibility testing and whole-genome sequencing.

**Results:**

A total of 144 patients were included. Hepatobiliary infection accounted for a majority of infections (73% [105 of 144]), which mostly occurred in elderly patients with comorbid conditions, including hepatobiliary complications. The all-cause 30-day mortality rate was 10.0% (95% confidence interval, 4.9%–14.8%). By whole-genome sequencing, 141 strains (98%) belonged to 4 *Aeromonas* species*—A caviae*, *A hydrophila*, *A veronii*, and *A dhakensis—*with significant intraspecies diversity. *A caviae* was predominant in all infection sites except skin and soft tissue, for which *A hydrophila* was the prevailing species. The genes encoding chromosomally mediated class B, C, and D β-lactamases were harbored by 92%–100% of the isolates in a species-specific manner, but they often lacked association with resistance phenotypes. The activity of cefepime was reliable. All isolates of *A hydrophila* and *A dhakensis* carried an *mcr-3-*like colistin resistance gene and showed reduced susceptibility to colistin.

**Conclusions:**

Hepatobiliary tract was the most common infection site of *Aeromonas* spp., with *A caviae* being the dominant causative species. The resistance genotype and phenotype were often incongruent for β-lactam agents.

## Teaser

The hepatobiliary tract was the most common infection site of *Aeromonas* spp., with *A caviae* the dominant causative species. The distribution of antimicrobial resistance genes was species specific, although the resistance genotype and phenotype were often incongruent for β-lactam agents.

The genus *Aeromonas* consists of gram-negative facultative anaerobic bacilli that are ubiquitously distributed in aquatic environments. *Aeromonas* spp. have been increasingly recognized as important pathogens not only to fish and other poikilothermic animals but also to humans [1], and they are linked to numerous human infectious diseases, ranging from mild illness, such as enterocolitis, to life-threatening conditions, including necrotizing fasciitis [1, 2]. Despite the growing clinical significance of *Aeromonas* infections, our understanding of them has been hampered by difficulty in accurate species identification based on standard microbiological techniques, even with the use of matrix-assisted laser desorption ionization-time of flight mass spectrometry [3, 4]. Furthermore, a significant change in the nomenclature over the past 2 decades complicated species-level identification process of this taxonomic group, now consisting of at least 30 *Aeromonas* species, including *A hydrophila*, *A caviae*, *A veronii*, and *A dhakensis,* which are mesophilic *Aeromonas* spp. frequently associated with human infections [1, 2, 5].

The complexity in speciation has also compromised our understanding of antimicrobial resistance pattern of this genus. Some *Aeromonas* species are intrinsically resistant to clinically important β-lactam agents through the expression of ≥2 discrete chromosomally mediated β-lactamases, including class B metallo-β-lactamases, class C cephalosporinases (AmpC), and class D oxacillinases (OXA) [1, 3]. These β-lactamases are known to be produced in unique combinations in each species, with their expression regulated by the 2-component regulatory system BlrAB [6–8]. However, species-level data on their prevalence and concordance with the resistance phenotype are still lacking. Furthermore, studies in recent years have suggested that some *Aeromonas* spp. harbor colistin resistance genes on their chromosome as potential sources of plasmid-mediated *mcr-3* and *mcr-7* in Enterobacterales [9–11].

We therefore aimed to investigate the clinical features of patients with diagnosed *Aeromonas* infections and to elucidate the genotypic and phenotypic characteristics of the causative bacterial strains, using whole-genome sequencing (WGS) data to support their taxonomic assignment. The species-specific antimicrobial resistance profiles were also evaluated, with the goal of informing treatment of patients with *Aeromonas* infections.

## METHODS

### Study Design and Population

This was a prospective multicenter cohort study of individuals with *Aeromonas* infection recruited at 6 tertiary hospitals in Japan between June 2020 and August 2022. The hospitals included 3 general hospitals, 2 university hospitals, and 1 cancer center. Patients were eligible for inclusion if *Aeromonas* spp. was isolated from a clinical culture and they were considered infected at the culture site, defined by standard criteria (Supplementary Materials). The strain must also have been available for further analysis. Cases deemed to represent only colonization were excluded. There was no exclusion based on age. During the study period, only the first episode of *Aeromonas* infection was included for each patient. The study was approved by the institutional review board at Fujita Health University Hospital (no. HM20-162) and all other participating hospitals. The opt-out recruitment method was used, and the requirement for informed consent was waived.

### Clinical Data Collection and Definition

The clinical data were collected from electronic medical records. For hepatobiliary infection, information on antimicrobial therapy was also collected. Detailed criteria and definitions used for each category are available in the Supplementary Materials.

### Microbiological Analysis

The strains were initially identified as *Aeromonas* spp. by the standard microbiological procedure at each hospital and were subsequently transported to the central research laboratory for further testing. Antimicrobial susceptibility was evaluated by means of broth microdilution, using a customized 96-well plate (Eiken Chemical) in accordance with the manufacturer's instructions. The results were interpreted according to the Clinical and Laboratory Standards Institute document M45 [12]. For antimicrobial agents without available breakpoints for *Aeromonas* spp. in that document, the 50th and 90th percentiles of the minimum inhibitory concentration (MIC) distributions (MIC_50_ and MIC_90_) and MIC ranges were presented.

### Species Identification and Molecular Analysis

All study isolates were subjected to WGS. Species-level identification was performed by calculating average nucleotide identity values against the corresponding type strain genome (Supplementary Table 1), with a cutoff value of 95% used for species delineation, combined with a phylogenetic analysis based on core-genome single-nucleotide polymorphisms. The details of genome sequencing and assembly, species identification, phylogenetic analysis, multilocus sequencing typing, and identification of antimicrobial resistance genes are available in the Supplementary Materials.

### Statistical Analysis

Categorical variables were compared with χ^2^ or Fisher exact tests, and continuous variables with Wilcoxon rank-sum test. Differences in the incidence of *Aeromonas* infections based on average monthly temperatures were evaluated using ordinal logistic regression models. Cumulative 14- and 30-day mortality rates were estimated using the Kaplan-Meier method. Detailed information on statistical analyses is available in the Supplementary Materials.

## RESULTS

### Patient Characteristics and Clinical Features

A total of 146 patients with *Aeromonas* infection were identified during the study period. After exclusion of 2 patients whose isolates were identified as species other than *Aeromonas* spp. by WGS analysis, 144 patients were included in the study. In this cohort, the incidence of *Aeromonas* infection was higher in months with an average monthly temperature >20°C (June–September) than in other months (odds ratio, 1.45 [95% confidence interval (CI), 1.09–1.93]; *P* = .01). The median patient age was 76 years (interquartile range, 70–83; range, 9 months to 98 years), and a majority of patients (84% [121 of 144]) were >65 years old. Male patients accounted for 59% (85 of 144), and the most frequent comorbid conditions were cancer (48% [69 of 144]), diabetes mellitus (16% [23 of 144]), and cerebrovascular diseases (14% [20 of 144]). Immunocompromised patients constituted 9% of the cohort, with a majority having a recent history of receiving chemotherapy.

The hepatobiliary tract was the most common site of *Aeromonas* infection, accounting for 73% of the cases (105 of 144), including cholangitis (84% [88 of 105]), cholecystitis (10% [11 of 105]), and liver abscess or infected liver cysts (7% [7 of 105]). Secondary bacteremia was observed in nearly half (49% [51 of 105]) of the patients with hepatobiliary infection. Other infections due to *Aeromonas* spp. included intra-abdominal infection (6.9% [10 of 144]), primary bacteremia (6.9% [10 of 144]), pneumonia (4.8% [7 of 144]), skin and soft-tissue infection (4.1% [6 of 144]), enterocolitis (2.6% [4 of 144]), and urinary tract infection (1.4% [2 of 144]). About half of the patients (46% [66 of 144]) acquired *Aeromonas* infection in the community. Details of the overall cohort are presented in Table 1.

**Table 1.** Demographic, Clinical, and Microbiological Characteristics of 144 Patients With *Aeromonas* Infections, Hepatobiliary or Nonhepatobiliary

Characteristic	Patients by Infection Type, No. (%)^a^			*P* Value
	All Infections (N = 144)	Hepatobiliary (n = 105)	Nonhepatobiliary (n = 39)	
Age, median (IQR), y	76 (70–83)	77 (70–83)	75 (61–83)	.35
Male sex	85 (59)	58 (55)	27 (69)	.13
Congestive heart failure	11 (8)	9 (9)	2 (5)	.49
Chronic lung diseases	8 (6)	7 (7)	1 (3)	.34
Connective tissue diseases	2 (1)	1 (1)	1 (3)	.46
Renal failure	8 (6)	7 (7)	1 (3)	.34
Diabetes mellitus	23 (16)	16 (15)	7 (18)	.69
Chronic liver disease	9 (6)	7 (7)	2 (5)	.73
Solid cancer	69 (48)	54 (51)	15 (39)	.17
Hepatobiliary	26 (18)	23 (22)	3 (8)	.049
Pancreatic	21 (15)	18 (17)	3 (8)	.15
Duodenal	6 (4)	4 (4)	2 (5)	.72
Other type	22 (15)	13 (12)	9 (23)	.11
Metastatic	31 (22)	24 (23)	7 (18)	.52
Cardiovascular disease	10 (7)	6 (6)	4 (10)	.34
Cerebrovascular disease	20 (14)	17 (16)	3 (8)	.19
Immunocompromised status	13 (9)	12 (11)	1 (3)	.10
Steroid therapy	1 (1)	1 (1)	0 (0)	.54
Chemotherapy	11 (8)	10 (10)	1 (3)	.16
Immunosuppressive therapy	1 (1)	1 (1)	0 (0)	.54
Hepatobiliary complications	102 (71)	87 (83)	15 (39)	<.001
Biliary obstruction/stricture	61 (42)	57 (54)	4 (10)	<.001
Due to stone	27 (19)	25 (24)	2 (5)	…
Due to tumor	29 (20)	27 (26)	2 (5)	…
Due to other causes	5 (4)	5 (5)	0 (0)	…
Cholelithiasis without blockage	5 (4)	3 (3)	2 (5)	.51
Artificial material	37 (26)	33 (31)	4 (10)	.01
Postoperative	42 (29)	32 (31)	10 (26)	.57
Others	6 (4)	5 (5)	1 (3)	.56
CCI, median (IQR)	6 (4.0–7.5)	6 (5.0–8.0)	5 (3.0–7.0)	.048
Type of acquisition				
Community acquired	66 (46)	49 (47)	17 (44)	.74
Healthcare associated	47 (33)	40 (38)	7 (18)	.02
Hospital acquired	31 (22)	16 (15)	15 (39)	.003
Length of hospital stay before diagnosis, median (IQR), d	7 (5–22)	7 (6–19)	12 (4–27)	.71
*Aeromonas* species				
*A caviae*	87 (60)	66 (63)	21 (54)	.33
*A hydrophila*	25 (17)	16 (15)	9 (23)	.27
*A veronii*	20 (14)	16 (15)	4 (10)	.44
*A dhakensis*	9 (6)	6 (6)	3 (8)	.66
*A allosaccharophila*	2 (1)	1 (1)	1 (3)	.46
*A media*	1 (1)	0 (0)	1 (3)	.10
Isolation from blood culture	66 (46)	51 (49)	15 (39)	.28
Species isolated from blood				
*A caviae*	39 (27)	30 (29)	9 (23)	.51
*A hydrophila*	11 (8)	8 (8)	3 (8)	.99
*A veronii*	9 (6)	8 (8)	1 (3)	.27
*A dhakensis*	7 (5)	5 (5)	2 (5)	.93
No. of coisolated pathogens				
1–2	66 (46)	48 (46)	18 (46)	.96
>3	41 (29)	31 (30)	10 (26)	.65
qSOFA score				
0–1	125 (87)	97 (92)	28 (72)	…
≥2	19 (13)	8 (7.6)	11 (28)	.001
Septic shock within 48 h	16 (11)	8 (7.6)	8 (21)	.03

Abbreviations: CCI, Charlson comorbidity index; IQR, interquartile range; qSOFA, quick sepsis-related organ failure assessment.

^a^Data represent no. (%) of patients unless otherwise specified.

Patients in each group were divided according to high and low qSOFA scores, and no comparison between two groups was made for the low scores, because it was not clinically relevant.

Given the high prevalence of hepatobiliary infection, univariate analysis was used to compare baseline characteristics between the patients with hepatobiliary infection and those with other infections. Those with hepatobiliary infection were more likely to have hepatobiliary complications (ie, biliary obstruction/stricture due to underlying cancer) than those without hepatobiliary infection (83% [87 of 105] vs 39% [15 of 39], respectively; *P* < .001) and had a higher median Charlson comorbidity index (interquartile range) (6 [5.0–8.0] vs 5 [3.0–7.0]; *P* = .048). No significant difference was observed in other underlying conditions.

In terms of causative species, *A caviae* was the most common species, accounting for 60% (87 of 144), followed by *A hydrophila* (17% [25 of 144]), *A veronii* (14% [20 of 144]), and *A dhakensis* (6% [9 of 144]). These 4 species composed 98% (141 of 144) of the entire cohort, with the remaining strains identified as *Aeromonas allosaccharophila* (n = 2) or *Aeromonas media* (n = 1). *Aeromonas* spp. were coisolated with other pathogenic bacteria in 74% of cases, primarily residents of the enteric flora such as *Escherichia coli*, *Klebsiella pneumoniae*, and *Enterococcus faecalis* (Supplementary Table 2). *A caviae* was the predominant causative species in all infection types except skin and soft-tissue infections, from which *A hydrophila* was most frequently recovered. More specifically, *A hydrophila* was found to differ in the composition of infection sites (*P* = .04), with skin and soft-tissue infections accounting for a significantly higher proportion (Supplementary Table 3).

The appropriateness of antimicrobial therapy was measured in patients with hepatobiliary infection, who composed a majority of this cohort. Of 105 patients, only 47 (46%) received appropriate antimicrobial therapy within 48 hours of diagnosis. This was primarily due to the use of β-lactam agents (eg, piperacillin-tazobactam) for empiric therapy, which were often inactive against the *Aeromonas* species isolated (Table 2). Nevertheless, a majority of patients (88%) underwent adequate source control procedures, and the overall mortality rates at day 14 and 30 were 1.9% (95% CI, 0%–4.5%) and 7.9% (2.5%–12.9%), respectively (Table 3). Meanwhile, patients with nonhepatobiliary infections were more acutely ill than those with hepatobiliary infection (quick sepsis-related organ failure assessment score, ≥2; 28% [11 of 39] vs 7.6% [8 of 105], respectively; *P* = .001), and their mortality rates at days 14 and 30 were higher, at 12.9% (95% CI, 1.7%–22.8%) and 15.5% (3.3%–26.2%), respectively. Among 6 patients with nonhepatobiliary infections who died within 30 days after diagnosis, 4 had primary bacteremia, 1 had pneumonia, and 1 had intra-abdominal infection. The 14-day and 30-day mortality rates for the overall cohort were 4.9% (95% CI, 1.3%–3.4%) and 10.0% (4.9%–14.8%), respectively.

**Table 2.** Antimicrobial Susceptibility Profiles of *Aeromonas* Species (*A caviae*, *A hydrophila*, *A*  *veronii*, and *A*  *dhakensis*) Isolated From Clinical Specimens

Antimicrobial Agent	*A caviae* (n = 87)				*A hydrophila* (n = 25)				*A veronii* (n = 20)				*A dhakensis* (n = 9)			
	MIC, μg/mL			Non-S, %^a^	MIC, μg/mL			Non-S, %^a^	MIC, μg/mL			Non-S, %^a^	MIC, μg/mL			Non-S, %^a^
	Range	MIC_50_	MIC_90_		Range	MIC_50_	MIC_90_		Range	MIC_50_	MIC_90_		Range	MIC_50_	MIC_90_	
SAM^b^	≤8/4 to >32/16	> 32/16	> 32/16	…	32/16 to >32/16	> 32/16	> 32/16	…	32/16 to >32/16	> 32/16	> 32/16	…	32/16 to >32/16	> 32/16	> 32/16	…
TZP	≤4/4 to >128/4	≤ 4/4	> 128/4	25	≤ 4/4 to >128/4	≤ 4/4	128/4	20	≤ 4/4 to >128/4	32/4	> 128/4	55	≤ 4/4 to >128/4	≤ 4/4	> 128/4	44
CTX	≤0.5 to >32	≤0.5	>32	41	≤0.5 to >32	≤0.5	2	16	≤0.5 to >32	≤0.5	≤0.5	5	≤0.5 to >32	8	>32	67
CAZ	≤0.5 to >32	≤0.5	>32	17	≤0.5 to >32	≤0.5	2	4	≤0.5 to >32	≤0.5	1	0	≤0.5 to >32	4	>32	44
ATM	≤4 to >32	≤4	≤4	6	≤4 to >32	≤4	≤4	4	≤4	≤4	≤4	0	≤4 to >32	≤4	>32	11
FEP	≤0.5–32	≤0.5	1	3	≤0.5–8	≤0.5	≤0.5	4	≤0.5–2	≤0.5	≤0.5	0	≤0.5–4	≤0.5	4	11
C/T^b^	≤2/4 to >16/4	≤ 2/4	≤ 2/4	…	≤ 2/4 to >16/4	≤ 2/4	≤ 2/4	…	≤ 2/4	≤ 2/4	≤ 2/4	…	≤ 2/4–16/4	≤ 2/4	16/4	…
IPM	≤0.25–2	≤0.25	0.5	1	0.25–8	2	4	60	≤0.25 to >16	2	>16	50	2 to >16	4	16	100
MEM	≤0.12–8	≤0.12	≤0.12	2	≤0.12–2	0.5	1	8	≤0.12 to >16	0.5	>16	15	≤0.12 to >16	1	>16	33
CIP	≤0.25 to >4	≤0.25	0.5	2	≤0.25 –>4	≤0.25	2	12	≤0.25–1	≤0.25	≤0.25	0	≤0.25–2	≤0.25	2	22
LVX	≤0.5–4	≤0.5	2	3	≤0.5 to >8	≤0.5	4	12	≤0.5 to >8	≤0.5	2	10	≤0.5–4	1	4	22
MIN	≤4–16	≤4	8	17	≤4 to >16	≤4	8	12	≤4–16	≤4	8	25	≤4–16	≤4	16	22
TGC^b^	≤0.5	≤0.5	≤0.5	…	≤0.5–1	≤0.5	≤0.5	…	≤0.5 to >8	≤0.5	≤0.5	…	≤0.5–1	≤0.5	1	…
CST^b^	≤0.25–2	0.5	0.5	…	≤0.25 to >4	>4	>4	…	≤0.25 to >4	0.5	0.5	…	>4 to >4	>4	>4	…
SXT	≤2/38 to >4/76	≤2/38	≤2/38	8	≤2/38	≤2/38	≤2/38	0	≤2/38	≤2/38	≤2/38	0	≤2/38 to >4/76	≤2/38	> 4/76	11
GEN	≤4–8	≤4	≤4	1	≤4	≤4	≤4	0	≤4–8	≤4	≤4	5	≤4	≤4	≤4	0
AMK	≤16–32	≤16	≤16	2	≤16–32	≤16	≤16	4	≤16–32	≤16	≤16	5	≤16–64	≤16	64	22
TOB^b^	≤4–8	≤4	≤4	…	≤4–16	≤4	8	…	≤4–8	≤4	8	…	≤4	≤4	≤4	…

Abbreviations: AMK, amikacin; ATM, aztreonam; C/T, ceftolozane-tazobactam; CAZ, ceftazidime; CIP, ciprofloxacin; CST, colistin; CTX, cefotaxime; FEP, cefepime; GEN, gentamicin; IPM, imipenem; LVX, levofloxacin; MIC, minimum inhibitory concentration; MIC_50_, 50th percentile of the MIC; MIC_90_, 90th percentile of the MIC; MEM, meropenem; MIN, minocycline; Non-S, nonsusceptible; SAM, ampicillin-sulbactam; SXT; trimethoprim-sulfamethoxazole; TGC, tigecycline; TOB, tobramycin; TZP, piperacillin-tazobactam.

^a^Isolates with intermediate and resistant testing results were grouped together into the Non-S category.

^b^Antimicrobial agents without available breakpoints in the Clinical and Laboratory Standards Institute document M45 [12].

### Genome Epidemiology of *Aeromonas* spp. Causing Human Infections

**Figure 1.**
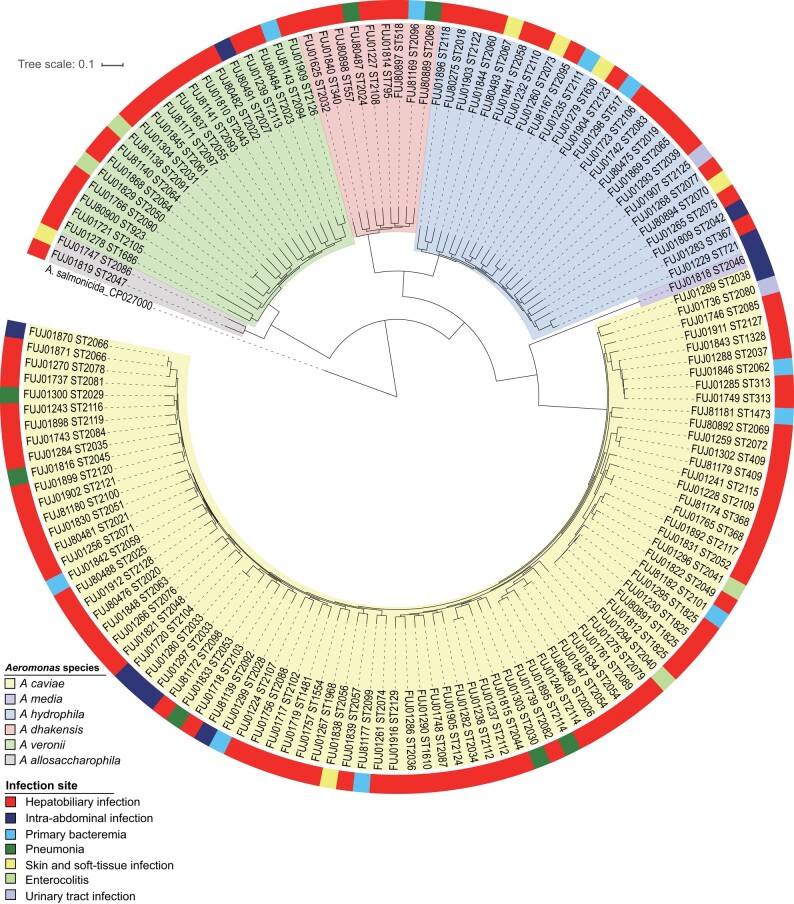
Core genome phylogeny of 144 *Aeromonas* isolates related to human infections. The nonduplicate isolate collected from each patient was included, and a maximum-likelihood phylogeny tree was constructed based on single-nucleotide polymorphisms (SNPs) identified in the core genomes of all isolates. The tree was rooted with a sequence from *Aeromonas salmonicida* (GenBank accession no. CP027000.1). The phylogeny was annotated based on the infection site, with sequence type (ST) listed next to the strain name. Branches are shaded by species described in the text.

A core-genome single-nucleotide polymorphism–based maximum likelihood tree was generated with nonduplicate isolates obtained from 144 patients to evaluate the phylogenetic relatedness of the study strains (Figure 1). The tree consisted of 4 major clades, corresponding to *A caviae*, *A hydrophila*, *A veronii*, and *A dhakensis*. The strains belonged to 132 discrete sequence types (STs), of which 20 were known STs and 112 were novel STs. Four *A caviae* ST1825 strains were recovered from unrelated individuals at 3 hospitals. Otherwise, a majority of the isolates belonged to unique STs, reflecting a highly diverse population of *Aeromonas* strains recovered in this study. No specific lineage was associated with particular infection site, including the hepatobiliary tract.

Next, to assess the genetic relatedness of the study strains and those collected from human sources in different geographic locations, we conducted a phylogenetic analysis using the genomes obtained in this study and publicly available *Aeromonas* genomes of human origin, deposited in GenBank as of 7 November 2022 (Supplementary Table 4), for 4 major *Aeromonas* species. A high degree of genetic diversity was observed in *Aeromonas* strains recovered from human sources, and there was no clustering of genomes correlating with particular geographic regions (Supplementary Figure).

### Antimicrobial Susceptibilities

The antimicrobial susceptibility patterns of 4 major *Aeromonas s*pp. are summarized in Table 2. Overall, cefepime, aztreonam, gentamicin, and trimethoprim-sulfamethoxazole demonstrated activity against 4 major *Aeromonas* species, with the susceptibility rates exceeding 89%. Fluoroquinolones were also active against a majority of the isolates, except for those belonging to *A dhakensis*, for which the susceptibility rate was slightly lower (78%). The susceptibility rates to β-lactam/β-lactamase inhibitors (eg, piperacillin-tazobactam), third-generation cephalosporins, and carbapenems were variable among the species, likely owing to the presence of chromosomally encoded β-lactamases, which are discussed below. Notably, the MICs of colistin were significantly higher for *A hydrophila* and *A dhakensis*, with MIC_50/_MIC_90_ values of >4/>4 μg/mL, compared with other species (*P* < .001).

**Table 3.** Treatment and Clinical Outcome of Patients With *Aeromonas* Infections, Hepatobiliary or Nonhepatobiliary

Parameter	Patients, No. (%)^a^	
	Hepatobiliary Infections (n = 105)	Nonhepatobiliary Infections (n = 39)
Antimicrobial therapy		
** **Appropriate antimicrobial agents started within 48 h	47 (46)	…
** **Appropriate antimicrobial agents not given within 48 h	55 (52)	…
** **Antimicrobial agents used for empirical therapy		
** **Piperacillin-tazobactam	17 (16)	…
** **Ampicillin-sulbactam	18 (17)	…
** **Cefoperazone-sulbactam	7 (7)	…
** **Cefmetazole	7 (7)	…
** **Others^b^	6 (6)	…
Presence of infection site requiring source control measure	81 (78)	…
Source control measure implemented	71 (88)	…
** **Within 24 h	47 (66)	…
** **In 24–48 h	12 (17)	…
** **After 48 h	12 (17)	…
ICU admission within 14 d	4 (3.8)	8 (21)
All-cause mortality rate (95% CI), %		
** **At 14 d	1.9 (0–4.5)	12.9 (1.7–22.8)
** **At 30 d	7.9 (2.5–12.9)	15.5 (3.3–26.2)

Abbreviations: CI, confidence interval; ICU, intensive care unit.

^a^Data represent no. (%) of patients unless otherwise specified.

^b^Others include flomoxef (n = 2), meropenem (n = 1), ceftriaxone (n = 1), cefepime (n = 1), and fosfomycin (n = 1).

### Antimicrobial Resistance Gene Profiles and Their Correlation With Resistance Phenotypes

**Figure 2.**
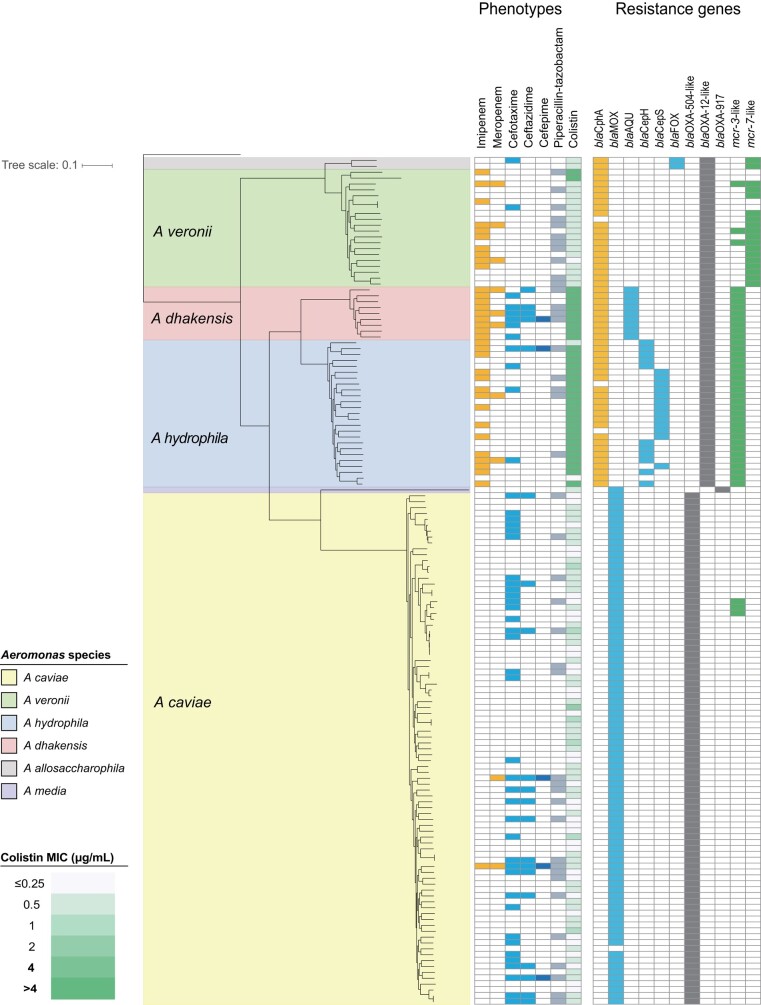
Phylogenetic tree of 144 *Aeromonas* isolates from Figure 1 shown with the susceptibility testing results and antimicrobial resistance genes. Nonsusceptibility testing results for carbapenems (imipenem and meropenem), third-generation cephalosporins (cefotaxime and ceftazidime), cefepime, and piperacillin-tazobactam are shown with orange, light blue, blue, and light gray boxes, respectively. For colistin, minimum inhibitory concentration (MIC) values are displayed with a color gradient, from white (low MICs) to green (high MICs). The presence of resistance genes is noted with colored boxes; orange represents *bla*_CphA_; blue, *bla*_MOX_, *bla*_CepH_/_CepS,_  *bla*_AQU_, and *bla*_FOX_; gray, *bla*_OXA-504-like,_  *bla*_OXA-12-like,_ and *bla*_OXA-917_; green, and *mcr-*like gene.

The presence of antimicrobial resistance genes and the antimicrobial susceptibility testing results are shown in Figure 2. A gene that chromosomally encodes a class B2 metallo-β-lactamase which efficiently hydrolyzes carbapenems [13], *bla*_CphA_, was harbored by 92%–100% of the isolates of *A hydrophila*, *A dhakensis,* and *A veronii*, while the discrete AmpC genes, *bla*_MOX_, *bla*_CepH_/_CepS,_ and *bla*_AQU,_ were detected in 96%–100% of the isolates of *A caviae*, *A hydrophila*, and *A dhakensis*, respectively. The incidences of these antimicrobial resistance genes in individual species were similar to those reported in previous studies [14, 15].

Despite the high prevalence of these β-lactamase genes, their concordance rates to the phenotypic resistance (ie, nonsusceptible testing results) were variable among species, ranging from 53% to 100% for *bla*_CphA_ and from 17% to 78% for *bla*_AmpC_ (Supplementary Table 5). For the isolates carrying *bla*_CphA,_ the resistance rate to imipenem was higher than that to meropenem, which was consistent with prior kinetic studies of CphA [16, 17]. Among 4 major species, *A dhakensis* showed the highest concordance for both *bla*_CphA_ and *bla*_AmpC_, corresponding to high rates of carbapenem and cephalosporin resistance (Table 2). The *bla*_OXA_ genes encoding OXA were present in all isolates in species-specific manner, and they were classified into 2 groups based on amino-acid sequence similarities: *bla*_OXA-504-like_ (*A caviae*) or *bla*_OXA-12-like_ (*A hydrophila*, *A dhakensis*, *A veronii*) [18]. It could not be determined whether the OXA enzymes affected the susceptibility to β-lactam/β-lactamase inhibitors [6, 19]. Genes encoding acquired β-lactamases, such as extended-spectrum β-lactamase or carbapenemase, were not detected in any of the study isolates.

The *mcr-3*-like genes, sharing 76%–78% amino acid sequence identity with *mcr*-*3.12*, were detected in all isolates belonging to *A hydrophila* and *A dhakensis* (n = 34). Of these isolates, 32 (94%) exhibited high MICs (>4 μg/mL) to colistin, demonstrating a strong correlation with this genotype. In contrast, while 80% of *A veronii* isolates 80% (16 of 20) of *A veronii* isolates harbored *mcr*-*7*-like genes sharing 70%–71% amino-acid sequence identity to *mcr-7.1*, all isolates exhibited low colistin MIC values (<0.5 μg/mL). These *mcr-*like genes were likely located on the chromosome. Information regarding other resistance determinants is available in Supplementary Table 6.

## DISCUSSION

*Aeromonas* spp. is increasingly recognized as a common human pathogen, but its population structure pertaining to human infections and their clinical implications are not well understood. The present study yielded several salient findings that serve to improve our understanding of this complex genus as a significant cause of invasive infections in humans.

We observed higher incidences of human *Aeromona*s infections during the warmer months of the year. This phenomenon, infection seasonality, has been well documented in mesophilic *Aeromonas* spp. and other gram-negative rods (eg, *Acinetobacter* spp.), which grow optimally at elevated temperatures [20, 21]. It is believed that the higher concentrations of these organisms in the environments during warmer months increase the likelihood of humans contracting them, although the details of the underlying mechanisms and transmission routes remain obscure [1, 21]. Hepatobiliary infection, particularly cholangitis, accounted for a majority of infections caused by *Aeromonas* spp. and tended to occur in elderly individuals with comorbid conditions, such as cancer and hepatobiliary complications.

These findings align with the epidemiological trend observed in East Asia, where a relatively high incidence of *Aeromonas* hepatobiliary infections has been reported, although not as high as observed in the present cohort (18% vs 73%, respectively) [15, 22, 23]. On the other hand, there were only few cases of skin and soft-tissue infection or enterocolitis, which are classically considered as the major clinical manifestations of *Aeromonas* spp. in the United States, Europe, and Australia [20, 24, 25]. Among the causative species, *A caviae* was predominant in most infection sites, including the hepatobiliary tract. The only exception was skin and soft tissues, where *A hydrophila* was isolated in significantly higher proportions, as described in the existing literature [15, 20, 26]. Overall, genetically diverse *Aeromonas* strains were involved in human infections, without any specific lineages representing hepatobiliary infection or distributed in specific geographic locations [27, 28]. These findings suggest that the unique epidemiological features observed in this study cannot be explained by specific *Aeromonas* clones circulating in the community. Instead, they are more likely attributable to a composite of various interactions among the pathogen, host, social, and environmental factors.

*Aeromonas* spp. are not part of the normal enteric flora of humans; however, transient colonization occurs following the consumption of contaminated foods or drinking water, with the fecal colonization rate ranging from 1% to 30% in asymptomatic individuals [1, 29]. Among various types of food, seafood (ie, fish and shellfish) has the highest contamination rate of *Aeromonas* spp. (72%–93%) [1, 29, 30]. Notably, studies revealed that 50%–70% of “ready to eat” raw fish (eg, sushi and sashimi), now widely consumed across the globe, contained *Aeromonas* spp. [31, 32]. It remains to be understood whether Japanese cuisine, characterized by frequent consumption of raw fish, is associated with increased risk of gastrointestinal colonization of *Aeromonas* spp. and, consequently, ascending bacterial infection in individuals with predisposing conditions. However, given the growing popularity of raw fish diet, the role of *Aeromonas* spp. as potential etiological agent of “foodborne illness” and their pathogenicity toward extraintestinal organs, particularly the hepatobiliary tract, may be speculated.

The genome-wide analysis of 144 clinical isolates also uncovered the species-specific distribution of antimicrobial resistance genes in *Aeromonas* spp., with chromosomal β-lactamase genes (*bla*_CphA,_  *bla*_AmpC_, and *bla*_OXA_) harbored by 92%–100% of the isolates within individual species. Nevertheless, their resistance genotypes and phenotypes were often incongruent, likely owing to variable expression of the enzymes [7, 8]. Prior studies suggested that additional tests (eg, susceptibility testing with a large inoculum, CarbaNP, or a modified carbapenem inactivation method (mCIM)) are required to detect CphA production [14, 33, 34]. Interestingly*, A dhakensis,* increasingly recognized as a pathogenic species to be related to a high mortality rate in bacteremic patients [35], exhibited the highest genotypic and phenotypic concordance for both *bla*_AmpC_ and *bla*_CphA_, corresponding to higher rates of carbapenem and cephalosporin resistance compared with other species.

Among β-lactam agents, the activity of cefepime was reliable in all species, which could be explained by its stability against both CphA and AmpC enzymes. Meanwhile, β-lactam/β-lactamase inhibitors showed variable susceptibility patterns, without apparent association with the presence or absence of OXA and AmpC enzymes. Although the information is scarce on how these intrinsic resistance mechanisms may affect clinical outcome of the patients with *Aeromonas* infection [15], the genotypic and phenotypic discordance should be considered when interpreting the susceptibility testing results and selecting treatment options for *Aeromonas* spp. infection, especially for critically ill patients.

As of today, 10 plasmid-mediated *mcr* genes (ie, *mcr-1* to *mcr-10*) have been described in various gram-negative bacilli worldwide, conferring reduced susceptibility to colistin [36]. Among them, *mcr-3* and *mcr-7* have been speculated to originate from *Aeromonas* spp., based on the presence of the *Aeromonas* chromosomal gene sequences that align closely with these *mcr* genes [9, 10]. However there have been conflicting data on the prevalence of *mcr* in individual *Aeromonas* spp. [11, 37], and the information on their colistin resistance has been scarce [11, 38]. In our study *mcr-3*-like genes were harbored in all isolates of *A hydrophila* and *A dhakensis*, with their presence correlating with high MICs of colistin, suggesting that these 2 species are intrinsically resistant to colistin. In contrast, *mcr-7*-like genes, detected in a majority of the *A veronii* strains, did not confer colistin resistance. This is in line with prior studies showing poor ability of MCR-7 to elicit colistin resistance, likely due to structural differences of the enzymes [39]. According to these findings, colistin is not recommended for the treatment of infection suspected or confirmed to be caused by *A hydrophila* and *A dhakensis*.

Our study has several limitations. First, it was a multicenter study conducted in Japan, and may not reflect trends in other geographic locations. Second, the limited number of *A dhakensis* isolates may have resulted in missed characteristics. Third, the majority of *Aeromonas* infections were polymicrobial, where the pathogenic role of each organism was difficult to be determined. Fourth, the clinical impact of infections caused by a strain exhibiting incongruent phenotypic and genotypic resistance could not be assessed owing to the small number of cases with outcomes. Fifth, WGS used for species identification in this study is still difficult to implement in general clinical settings.

In summary, hepatobiliary infection was the most common clinical manifestation of human *Aeromonas* infections, with *A caviae* being the predominant causative species. The prevalence of antimicrobial resistance genes was species specific, and resistance genotype and phenotype were often incongruent for β-lactams, which should be considered when interpreting the susceptibility testing results and selecting antimicrobial agents for therapy.

## Supplementary Material

ofad587_Supplementary_Data

## Supplementary Data

Supplementary materials are available at *Open Forum Infectious Diseases* online. Consisting of data provided by the authors to benefit the reader, the posted materials are not copyedited and are the sole responsibility of the authors, so questions or comments should be addressed to the corresponding author.
