# Supplementary material for: Clinical Features, Genome Epidemiology, and Antimicrobial Resistance Profiles of Aeromonas spp. Causing Human Infections: A Multicenter Prospective Cohort Study
Source: Open Forum Infect Dis. 2023 Nov 16;10(12):ofad587. doi: 10.1093/ofid/ofad587 (PMC10753922; doi:10.1093/ofid/ofad587)
Supplement: ofad587_Supplementary_Data [file ofad587_supplementary_data.zip › Supp_Table_1_new.docx]

**Supplementary Table 1.** Reference genomes representing 30 species of the genus A*eromonas.*

| *Aeromonas* species | Taxonomy ID | Accession no. | BioSample | BioProject | Strain | Level | Size (Mb) | GC% | Author citation (author(s)-year) |
| --- | --- | --- | --- | --- | --- | --- | --- | --- | --- |
| *A. allosaccharophila* | 656 | NKWZ01 | SAMN07312751 | PRJNA391781 | ATCC 35942^T^ | Scaffold | 4.5 | 59 | Martinez-Murcia et al. 1992 |
| *A. aquatica* | 558964 | JRGL01 | SAMN03023875 | PRJNA260478 | AE235^T^ | Contig | 4.6 | 61 | Beaz-Hidalgo et al. 2015 |
| *A. australiensis* | 1114880 | CDDH01 | SAMEA2752426 | PRJEB7021 | CECT 8023^T^ | Contig | 4.1 | 58 | Aravena-Román et al. 2013 |
| *A. bestiarum* | 105751 | CDDA01 | SAMEA2752425 | PRJEB7022 | CECT 4227^T^ | Scaffold | 4.7 | 61 | Ali et al. 1996 |
| *A. bivalvium* | 440079 | CDBT01 | SAMEA2752424 | PRJEB7023 | CECT 7113^T^ | Scaffold | 4.3 | 62 | Miñana-Galbis et al. 2007 |
| *A. caviae* | 648 | LS483441.1 | SAMEA4475690 | PRJEB6403 | NCTC12244 | Complete | 4.6 | 62 | Popoff 1984 |
| *A. dhakensis* | 196024 | CDBH01 | SAMEA2752400 | PRJEB7048 | CIP 107500^T^ | Scaffold | 4.7 | 62 | Beaz-Hidalgo et al. 2015 |
| *A. diversa* | 502790 | CDCE01 | SAMEA2752422 | PRJEB7026 | CECT 4254^T^ | Scaffold | 4.1 | 62 | Miñana-Galbis et al. 2010 |
| *A. encheleia* | 73010 | LR134376.1 | SAMEA4475689 | PRJEB6403 | NCTC12917^T^ | Complete | 4.5 | 62 | Esteve et al. 1995 |
| *A. enteropelogenes* | 29489 | CDCG01 | SAMEA2752420 | PRJEB7028 | CECT 4487^T^ | Scaffold | 4.5 | 60 | Schubert et al. 1991 |
| *A. eucrenophila* | 649 | CDDF01 | SAMEA2752419 | PRJEB7029 | CECT 4224^T^ | Scaffold | 4.5 | 61 | Schubert and Hegazi 1988 |
| *A. finlandensis* | 1543375 | JRGK01 | SAMN03023686 | PRJNA260478 | 4287D^T^ | Contig | 4.7 | 59 | Beaz-Hidalgo et al. 2015 |
| *A. fluvialis* | 591962 | CDBO01 | SAMEA2752418 | PRJEB7030 | CDBO01^T^ | Scaffold | 3.9 | 58.3 | Alperi et al. 2010 |
| *A. hydrophila* | 380703 | CP000462.1 | SAMN02604052 | PRJNA16697 | ATCC7966^T^ | Complete | 4.7 | 62 | Stanier 1943 |
| *A. jandaei* | 650 | CDBV01 | SAMEA2752417 | PRJEB7031 | CECT 4228^T^ | Scaffold | 4.5 | 59 | Carnahan et al. 1992 |
| *A. lacus* | 558884 | JRGM01 | SAMN03023876 | PRJNA260478 | AE122^T^ | Contig | 4.4 | 59 | Beaz-Hidalgo et al. 2015 |
| *A. media* | 651 | CDBZ01 | SAMEA2752416 | PRJEB7032 | CECT 4232^T^ | Scaffold | 4.5 | 61 | Allen et al. 1983 |
| *A. molluscorum* | 271417 | AQGQ01 | SAMN02471397 | PRJNA183610 | 848^T^ | Contig | 4.2 | 59 | Miñana-Galbis et al. 2004 |
| *A. piscicola* | 600645 | CDBL01 | SAMEA2752415 | PRJEB7033 | LMG24783^T^ | Scaffold | 5.2 | 59 | Beaz-Hidalgo et al. 2010 |
| *A. popoffii* | 70856 | CDBI01 | SAMEA2752414 | PRJEB7034 | CIP105493^T^ | Scaffold | 4.8 | 59 | Huys et al. 1997 |
| *A. rivipollensis* | 948519 | CP027856.1 | SAMN08721782 | PRJNA438570 | KN-Mc-11N1^T^ | Complete | 4.5 | 62 | Marti and Balcázar 2016 |
| *A. rivuli* | 648794 | CDBJ01 | SAMEA2752413 | PRJEB7035 | DSM 22539^T^ | Scaffold | 4.5 | 60 | Figueras et al. 2011 |
| *A. salmonicida* | 29491 | CP027000.1 | SAMN02469939 | PRJNA264317 | 01-B526^T^ | Complete | 4.7 | 58 | Griffin et al. 1953 |
| *A. sanarellii* | 633415 | CDBN01 | SAMEA2752411 | PRJEB7037 | LMG 24682^T^ | Scaffold | 4.2 | 63 | Alperi et al. 2010 |
| *A. schubertii* | 652 | CDDB01 | SAMEA2752410 | PRJEB7038 | CECT 4240^T^ | Scaffold | 4.1 | 62 | Hickman-Brenner et al. 1989 |
| *A. simiae* | 218936 | CDBY01 | SAMEA2752409 | PRJEB7039 | CIP 107798^T^ | Scaffold | 4.0 | 61 | Harf-Monteil et al. 2004 |
| *A. sobria* | 646 | CDBW01 | SAMEA2752408 | PRJEB7040 | CECT 4245^T^ | Scaffold | 4.7 | 58 | Popoff and Véron 1981 |
| *A. taiwanensis* | 633417 | CDDD01 | SAMEA2752407 | PRJEB7041 | LMG 24683^T^ | Scaffold | 4.3 | 63 | Alperi et al. 2010 |
| *A. tecta* | 324617 | CDCA01 | SAMEA2752406 | PRJEB7042 | CDCA01^T^ | Scaffold | 4.8 | 60.1 | Demarta et al. 2010 |
| *A. veronii* | 197701 | CDDK01 | SAMEA2752404 | PRJEB7044 | CECT4257^T^ | Scaffold | 4.5 | 59 | Hickman-Brenner et al. 1988 |
