# Supplementary material for: Clinical Features, Genome Epidemiology, and Antimicrobial Resistance Profiles of Aeromonas spp. Causing Human Infections: A Multicenter Prospective Cohort Study
Source: Open Forum Infect Dis. 2023 Nov 16;10(12):ofad587. doi: 10.1093/ofid/ofad587 (PMC10753922; doi:10.1093/ofid/ofad587)
Supplement: ofad587_Supplementary_Data [file ofad587_supplementary_data.zip › Supp_Table_2.docx]

**Supplementary Table 2.** Pathogenic bacteria co-isolated with *Aeromonas* spp. from clinical specimens, stratified into those with and without hepatobiliary infections.

|  | Total  (n=144) | Hepatobiliary infections  (n=105) | Non-hepatobiliary infections  (n=39) | |
| --- | --- | --- | --- | --- |
| *Escherichia coli* (%) | 35 (24) | 24 (23) | 11 (28) | |
| *Klebsiella pneumoniae* (%) | 33 (23) | 26 (25) | 7 (18) | |
| *Klebsiella oxytoca* (%) | 21 (15) | 17 (16) | 4 (10) | |
| *Enterococcus faecalis* (%) | 21 (15) | 15 (14) | 6 (15) | |
| *Enterococcus faecium* (%) | 16 (11) | 14 (13) | | 2 (5) |
| Other *Enterococcus* spp. (%) | 16 (11) | 15 (14) | 1 (3) | |
| *Citrobacter* spp. (%) | 15 (10) | 14 (13) | 1 (3) | |
| *Clostridium perfringens* (%) | 11 (8) | 11 (11) | 0 (0) | |
| *Enterobacter* spp. (%) | 10 (7) | 7 (7) | 3 (8) | |
| *Klebsiella aerogenes* (%) | 8 (6) | 3 (3) | 5 (13) | |
| *Morganella morganii* (%) | 10 (7) | 7 (7) | 3 (8) | |
| *Candida* spp. (%) | 4 (3) | 2 (2) | 2 (5) | |
| Others* (%) | 34 (24) | 23 (22) | 11 (28) | |

*Others include *Pseudomonas* spp.***,*** *Staphylococcus aureus, Streptococcus* spp.***,*** *Proteus* spp**.,** *Fusobacterium* spp**.,** *Serratia* spp.**,** *Raultella ornitholytica****,*** *Shewanella algae****,*** *Stenotrohomoans maltophilia****,*** *Acinetobacter* spp.***,*** *Veionella prrvula****,*** *Biophila wadsworthia****,*** *Prevotella buccae****,*** *Bacillus* spp**.,** *Haemophilus parainfuenzae****,*** *Salmonella* spp.***,*** *Campylobacter* spp**.,** *Hafnia alvei****,*** *Actinomyces odontolyticus****,*** *Lactobacillus lactis****,*** *Slackia exigua****,*** *Klebsiella variicola.* Data are presented as No. (%).
