# Supplementary material for: Clinical Features, Genome Epidemiology, and Antimicrobial Resistance Profiles of Aeromonas spp. Causing Human Infections: A Multicenter Prospective Cohort Study
Source: Open Forum Infect Dis. 2023 Nov 16;10(12):ofad587. doi: 10.1093/ofid/ofad587 (PMC10753922; doi:10.1093/ofid/ofad587)
Supplement: ofad587_Supplementary_Data [file ofad587_supplementary_data.zip › Supp_Table_3.docx]

**Supplementary Table 3.** *Aeromonas* species isolated from each infection site.

| Infection site | Number  of cases | Causative species [number of cases (%)] | | | | | |
| --- | --- | --- | --- | --- | --- | --- | --- |
|  |  | *A. caviae* | *A. hydrophila* | *A. veronii* | *A. dhakensis* | *A. allosaccharophila* | *A. media* |
| Hepatobiliary tract  [cholangitis, 88 (84%);  cholecystitis, 11 (10%);  liver abscess /infected liver cyst, 7 (7%)] | 105 | 66 (63%) | 16 (15%) | 16 (15%) | 6 (5.7%) | 1 (0.95%) | 0 |
| Intra-abdomen  [organ-space SSI, 7 (70%);  peritonitis, 2 (20%); other, 1 (10%)] | 10 | 6 (60%) | 2 (20%) | 1 (10%) | 0 | 0 | 1 (10%) |
| Primary bacteremia | 10 | 6 (60%) | 2 (20%) | 1 (10%) | 1 (10%) | 0 | 0 |
| Respiratory tract  [pneumonia, 7 (100%)] | 7 | 5 (71%) | 0 | 0 | 2 (29%) | 0 | 0 |
| Skin and soft tissue  [SSI, 2 (33%); non-SSI, 4 (67%)] | 6 | 1 (17%) | 4 (67%) | 0 | 0 | 1 (17%) | 0 |
| Gastrointestinal tract  [enterocolitis, 4 (100%)] | 4 | 2 (50%) | 0 | 2(50%) | 0 | 0 | 0 |
| Urinary tract | 2 | 1 (50%) | 1 (50%) | 0 | 0 | 0 | 0 |
| P-value | - | 0.435 | 0.039 | 0.445 | 0.309 | - | - |

Abbreviation: SSI, surgical site infection. The difference in species distribution among infection sites was evaluated by frequency and Fisher's exact test.
