# Supplementary material for: Clinical Features, Genome Epidemiology, and Antimicrobial Resistance Profiles of Aeromonas spp. Causing Human Infections: A Multicenter Prospective Cohort Study
Source: Open Forum Infect Dis. 2023 Nov 16;10(12):ofad587. doi: 10.1093/ofid/ofad587 (PMC10753922; doi:10.1093/ofid/ofad587)
Supplement: ofad587_Supplementary_Data [file ofad587_supplementary_data.zip › Supp_Table_4.docx]

**Supplementary Table 4**. *Aeromonas* genomes registered in GenBank, isolated from human sources.

| Organism | BioSample | Accession  No. | BioProject | Isolation  source | Geographic  location | Strain | Level | Size  (Mb) | GC  (%) | Release  Date |
| --- | --- | --- | --- | --- | --- | --- | --- | --- | --- | --- |
| *A. caviae* | SAMN29408258 | CP100392 | PRJNA853890 | bile | China:Zhengzhou | FAHZZU2447 | Complete | 4.8 | 61 | 2022-07-12 |
| *A. caviae* | SAMN07680230 | NXBW01 | PRJNA408193 | rectal swab | China | ZJ33-3 | Contig | 4.5 | 62 | 2018-06-26 |
| *A. caviae* | SAMN13683804 | WUTZ01 | PRJNA597794 | Watery diarrhea | China: Luzhou | ScAc2001 | Contig | 4.5 | 61 | 2020-01-06 |
| *A. caviae* | SAMN13763658 | JAAALV01 | PRJNA600002 | wound | NA | BVH98 | Scaffold | 4.5 | 61 | 2020-01-19 |
| *A. caviae* | SAMN13763657 | JAAALW01 | PRJNA600002 | stool | NA | BVH84 | Scaffold | 4.5 | 61 | 2020-01-19 |
| *A. caviae* | SAMN13763656 | JAAALX01 | PRJNA600002 | intra-abdominal hematoma | NA | ADV118 | Scaffold | 4.4 | 62 | 2020-01-19 |
| *A. caviae* | SAMN14272287 | JAAROF01 | PRJNA612546 | feces | Mexico | 6597 | Scaffold | 4.5 | 61 | 2022-04-22 |
| *A. caviae* | SAMN03280160 | LESK01 | PRJNA271899 | missing | USA | BWH65 | Scaffold | 4.4 | 62 | 2015-06-19 |
| *A. caviae* | SAMN10457758 | RQJY01 | PRJNA506284 | fecal sample | Brazil: Sao Bento do Una | Aer268 | Scaffold | 4.5 | 61 | 2018-12-03 |
| *A. caviae* | SAMEA110089369 | CALTYR01 | PRJEB47281 | human skin | USA | jhKbiEa5yl_bin.62.MAG | Contig | 4.9 | 62 | 2022-06-13 |
| *A. hydrophila* | SAMN05589911 | CP016990 | PRJNA339336 | Wound secretion | China | ZYAH75 | Complete | 5.0 | 61 | 2018-09-07 |
| *A. hydrophila* | SAMN21168046 | CP084353 | PRJNA759416 | blood | China:Ningbo | 71317 | Complete | 4.7 | 62 | 2021-10-10 |
| *A. hydrophila* | SAMN21168048 | CP084352 | PRJNA759416 | urine | China:Ningbo | 71339 | Complete | 4.8 | 61 | 2021-10-10 |
| *A. hydrophila* | SAMN26095833 | CP092708 | PRJNA806525 |  | Sudan | S-P-C-021.01 | Chromosome | 4.8 | 62 | 2022-03-06 |
| *A. hydrophila* | SAMN26095834 | CP092709 | PRJNA806525 |  | Sudan | S-P-C-022.01 | Chromosome | 4.8 | 62 | 2022-03-06 |
| *A. hydrophila* | SAMN02471855 | AOBO01 | PRJNA183199 | pus | Malaysia | 187 | Contig | 4.8 | 62 | 2013-04-06 |
| *A. hydrophila* | SAMN02471868 | AOBP01 | PRJNA183201 | blood | Malaysia | 259 | Contig | 4.7 | 62 | 2013-04-06 |
| *A. hydrophila* | SAMN15641156 | JACLAM01 | PRJNA648413 | T-tube Fluid | USA | B-2 | Contig | 5.2 | 61 | 2020-09-02 |
| *A. hydrophila* | SAMN15641139 | JACLAO01 | PRJNA648413 | blood | USA | A-1 | Contig | 5.0 | 61 | 2020-09-02 |
| *A. hydrophila* | SAMN17083345 | JAEHHP01 | PRJNA685342 | stool | China: Shaanxi | CN17A0062 | Contig | 4.7 | 62 | 2021-01-20 |
| *A. hydrophila* | SAMN17083342 | JAEHHS01 | PRJNA685342 | stool | China: Shaanxi | CN17A0055 | Contig | 4.7 | 62 | 2021-01-20 |
| *A. hydrophila* | SAMN17083375 | JAEHIT01 | PRJNA685342 | stool | China: Shenzhen | CN17A0136 | Contig | 4.9 | 61 | 2021-01-20 |
| *A. hydrophila* | SAMN17083374 | JAEHIU01 | PRJNA685342 | stool | China: Shenzhen | CN17A0135 | Contig | 4.7 | 61 | 2021-01-20 |
| *A. hydrophila* | SAMN17083373 | JAEHIV01 | PRJNA685342 | stool | China: Shenzhen | CN17A0134 | Contig | 4.7 | 62 | 2021-01-20 |
| *A. hydrophila* | SAMN17083349 | JAEHJS01 | PRJNA685342 | stool | China: Shenzhen | CN17A0078 | Contig | 4.7 | 62 | 2021-01-20 |
| *A. hydrophila* | SAMN21168047 | JAJDSS01 | PRJNA759416 | pus | China:Ningbo | 71328 | Contig | 4.9 | 61 | 2021-10-26 |
| *A. hydrophila* | SAMN02597476 | JDWB01 | PRJNA237913 | clinical | USA | NF1 | Contig | 4.8 | 61 | 2014-06-16 |
| *A. hydrophila* | SAMN02597477 | JDWC01 | PRJNA237917 | clinical | USA | NF2 | Contig | 4.8 | 61 | 2014-06-16 |
| *A. hydrophila* | SAMN02643439 | JEMK01 | PRJNA183197 | human pus | Malaysia: Kuala Lumpur | 145 | Contig | 4.9 | 62 | 2014-03-07 |
| *A. hydrophila* | SAMN02643440 | JEML01 | PRJNA183200 | human Urine | Malaysia: Kuala Lumpur | 226 | Contig | 5.1 | 61 | 2014-03-07 |
| *A. hydrophila* | SAMN27512291 | JALKAG01 | PRJNA823662 | cerebrospinal fluid | China | Ah2101 | Scaffold | 5.2 | 61 | 2022-04-16 |
| *A. hydrophila* | SAMN03197759 | JVCD01 | PRJNA267549 | missing | USA: WA | 56_AHYD | Scaffold | 4.7 | 62 | 2015-07-10 |
| *A. hydrophila* | SAMN03197725 | JVDL01 | PRJNA267549 | missing | USA: WA | 53_AHYD | Scaffold | 4.7 | 62 | 2015-07-10 |
| *A. hydrophila* | SAMN03197714 | JVDW01 | PRJNA267549 | missing | USA: WA | 52_AHYD | Scaffold | 4.7 | 62 | 2015-07-10 |
| *A. hydrophila* | SAMN03197692 | JVES01 | PRJNA267549 | missing | USA: WA | 50_AHYD | Scaffold | 4.7 | 62 | 2015-07-10 |
| *A. hydrophila* | SAMN03197672 | JVFM01 | PRJNA267549 | missing | USA: WA | 48_AHYD | Scaffold | 4.7 | 62 | 2015-07-10 |
| *A. hydrophila* | SAMN03742454 | LSZC01 | PRJNA297179 | wound isolate | USA: California | AH1 | Scaffold | 4.8 | 61 | 2017-12-12 |
| *A. hydrophila* | SAMN10457754 | RQKC01 | PRJNA506284 | fecal sample | Brazil: Sao Bento do Una | Aer284 | Scaffold | 4.8 | 61 | 2018-11-28 |
| *A. dhakensis* | SAMN15184665 | CP054854 | PRJNA638258 | drainage | China: Beijing | 1706-28330 | Complete | 4.9 | 62 | 2021-03-09 |
| *A. dhakensis* | SAMN21168049 | CP084351 | PRJNA759416 | bile | China:Ningbo | 71431 | Complete | 4.8 | 62 | 2021-10-10 |
| *A. dhakensis* | SAMN21168051 | CP084349 | PRJNA759416 | bile | China:Ningbo | 71453 | Complete | 4.9 | 61 | 2021-10-10 |
| *A. dhakensis* | SAMN02471842 | AOBN01 | PRJNA183198 | peritoneal fluid | Malaysia | 173 | Contig | 4.8 | 62 | 2013-04-06 |
| *A. dhakensis* | SAMN02471843 | AOBQ01 | PRJNA183202 | pus | Malaysia | 277 | Contig | 4.8 | 62 | 2013-04-06 |
| *A. dhakensis* | SAMN17083340 | JAEHHU01 | PRJNA685342 | stool | China: Anhui | CN17A0050 | Contig | 4.8 | 62 | 2021-01-20 |
| *A. dhakensis* | SAMN17083384 | JAEHIK01 | PRJNA685342 | stool | China: Hebei | CN17A0194 | Contig | 4.7 | 62 | 2021-01-20 |
| *A. dhakensis* | SAMN17083378 | JAEHIQ01 | PRJNA685342 | stool | China: Shenzhen | CN17A0164 | Contig | 4.7 | 62 | 2021-01-20 |
| *A. dhakensis* | SAMN17083370 | JAEHIY01 | PRJNA685342 | stool | China: Shenzhen | CN17A0124 | Contig | 4.8 | 61 | 2021-01-20 |
| *A. dhakensis* | SAMN17083359 | JAEHJI01 | PRJNA685342 | stool | China: Shenzhen | CN17A0101 | Contig | 4.9 | 61 | 2021-01-20 |
| *A. dhakensis* | SAMN17083358 | JAEHJJ01 | PRJNA685342 | stool | China: Shenzhen | CN17A0100 | Contig | 4.8 | 62 | 2021-01-20 |
| *A. dhakensis* | SAMN17083348 | JAEHJT01 | PRJNA685342 | stool | China: Shenzhen | CN17A0075 | Contig | 4.8 | 62 | 2021-01-20 |
| *A. dhakensis* | SAMN17083329 | JAEIJA01 | PRJNA685342 | stool | China: Anhui | CN17A0022 | Contig | 4.8 | 62 | 2021-01-20 |
| *A. dhakensis* | SAMN17083328 | JAEIJB01 | PRJNA685342 | stool | China: Anhui | CN17A0014 | Contig | 4.7 | 62 | 2021-01-20 |
| *A. dhakensis* | SAMN17101842 | JAGDET01 | PRJNA685948 | Exudate from surgical wound | Spain: Catalonia | 547_SP | Contig | 4.8 | 62 | 2021-05-14 |
| *A. dhakensis* | SAMN02597481 | JDWD01 | PRJNA237907 | clinical | Philippines | SSU | Contig | 4.9 | 62 | 2014-06-16 |
| *A. dhakensis* | SAMN10457753 | RQKD01 | PRJNA506284 | fecal sample | Brazil: Sao Bento do Una | Aer283 | Contig | 4.7 | 62 | 2018-11-28 |
| *A. dhakensis* | SAMN02463947 | AGWR01 | PRJNA71509 |  | not determined | SSU | Scaffold | 4.9 | 62 | 2012-09-17 |
| *A. dhakensis* | SAMN17860435 | JAGFDX01 | PRJNA701275 | urine | Nigeria | 15996 | Scaffold | 4.8 | 62 | 2021-03-24 |
| *A. dhakensis* | SAMN17860440 | JAGFEC01 | PRJNA701275 | urine | Nigeria | 16006 | Scaffold | 4.9 | 62 | 2021-03-24 |
| *A. dhakensis* | SAMN07312764 | NKWO01 | PRJNA391781 | missing | Martinique | BVH70 | Scaffold | 4.7 | 62 | 2019-06-11 |
| *A. dhakensis* | SAMN07312763 | NKWP01 | PRJNA391781 | missing | Martinique | BVH69 | Scaffold | 4.8 | 62 | 2019-06-11 |
| *A. dhakensis* | SAMN07312762 | NKWQ01 | PRJNA391781 | missing | Martinique | BVH68 | Scaffold | 4.9 | 62 | 2019-06-11 |
| *A. dhakensis* | SAMN07312758 | NKWT01 | PRJNA391781 | wound | not available | BVH43 | Scaffold | 5.0 | 61 | 2019-06-11 |
| *A. dhakensis* | SAMN07312761 | PDXI01 | PRJNA391781 | blood | not available | BVH65 | Scaffold | 4.8 | 62 | 2019-06-10 |
| *A. dhakensis* | SAMN09770288 | QUOJ01 | PRJNA484846 | blood | China | 17FW001 | Scaffold | 4.7 | 62 | 2018-08-22 |
| *A. dhakensis* | SAMN10390361 | RJCW01 | PRJNA504324 | blood culture | Australia: Brisbane | AE-13 | Scaffold | 4.7 | 62 | 2018-11-13 |
| *A. veronii* | SAMN18498901 | CP072325 | PRJNA717373 | blood | China: Hebei | 183026 | Complete | 4.6 | 59 | 2022-06-27 |
| *A. veronii* | SAMN15184730 | CP054855 | PRJNA638265 | bile | China: Beijing | 1708-29120 | Complete | 4.5 | 59 | 2021-03-09 |
| *A. veronii* | SAMN15587301 | JACEGL01 | PRJNA525849 | blood | Thailand:Suratthani | C198 | Contig | 4.6 | 59 | 2020-07-26 |
| *A. veronii* | SAMN17083346 | JAEHHO01 | PRJNA685342 | stool | China: Shaanxi | CN17A0067 | Contig | 4.5 | 59 | 2021-01-20 |
| *A. veronii* | SAMN17083344 | JAEHHQ01 | PRJNA685342 | stool | China: Shaanxi | CN17A0059 | Contig | 4.3 | 59 | 2021-01-20 |
| *A. veronii* | SAMN17083341 | JAEHHT01 | PRJNA685342 | stool | China: Shaanxi | CN17A0054 | Contig | 4.3 | 59 | 2021-01-20 |
| *A. veronii* | SAMN17083339 | JAEHHV01 | PRJNA685342 | stool | China: Anhui | CN17A0049 | Contig | 4.3 | 59 | 2021-01-20 |
| *A. veronii* | SAMN17083337 | JAEHHX01 | PRJNA685342 | stool | China: Anhui | CN17A0040 | Contig | 4.4 | 59 | 2021-01-20 |
| *A. veronii* | SAMN17083335 | JAEHHZ01 | PRJNA685342 | stool | China: Anhui | CN17A0036 | Contig | 4.5 | 59 | 2021-01-20 |
| *A. veronii* | SAMN17083334 | JAEHIA01 | PRJNA685342 | stool | China: Anhui | CN17A0031 | Contig | 4.4 | 59 | 2021-01-20 |
| *A. veronii* | SAMN17083333 | JAEHIB01 | PRJNA685342 | stool | China: Anhui | CN17A0029 | Contig | 4.6 | 59 | 2021-01-20 |
| *A. veronii* | SAMN17083327 | JAEHIF01 | PRJNA685342 | stool | China: Anhui | CN17A0013 | Contig | 4.5 | 59 | 2021-01-20 |
| *A. veronii* | SAMN17083369 | JAEHIZ01 | PRJNA685342 | stool | China: Shenzhen | CN17A0122 | Contig | 4.5 | 59 | 2021-01-20 |
| *A. veronii* | SAMN17083364 | JAEHJD01 | PRJNA685342 | stool | China: Shenzhen | CN17A0114 | Contig | 4.4 | 59 | 2021-01-20 |
| *A. veronii* | SAMN17083361 | JAEHJG01 | PRJNA685342 | stool | China: Shenzhen | CN17A0103 | Contig | 4.4 | 59 | 2021-01-20 |
| *A. veronii* | SAMN17083360 | JAEHJH01 | PRJNA685342 | stool | China: Shenzhen | CN17A0102 | Contig | 4.5 | 59 | 2021-01-20 |
| *A. veronii* | SAMN17083357 | JAEHJK01 | PRJNA685342 | stool | China: Shenzhen | CN17A0097 | Contig | 4.5 | 59 | 2021-01-20 |
| *A. veronii* | SAMN17083355 | JAEHJM01 | PRJNA685342 | stool | China: Shenzhen | CN17A0093 | Contig | 4.3 | 59 | 2021-01-20 |
| *A. veronii* | SAMN17083353 | JAEHJO01 | PRJNA685342 | stool | China: Shenzhen | CN17A0087 | Contig | 4.6 | 59 | 2021-01-20 |
| *A. veronii* | SAMN17083368 | JAEHOJ01 | PRJNA685342 | stool | China: Shenzhen | CN17A0120 | Contig | 4.5 | 59 | 2021-01-20 |
| *A. veronii* | SAMN17101839 | JAGDEQ01 | PRJNA685948 | sputum | USA: Michigan | ATCC 35624 | Contig | 4.5 | 59 | 2021-05-14 |
| *A. veronii* | SAMN17101845 | JAGDEW01 | PRJNA685948 | feces | Spain: Catalonia | 551_SP | Contig | 4.6 | 59 | 2021-05-14 |
| *A. veronii* | SAMN17101847 | JAGDEY01 | PRJNA685948 | feces | Spain: Catalonia | 553_SP | Contig | 4.6 | 59 | 2021-05-14 |
| *A. veronii* | SAMN20667228 | JAIEXX01 | PRJNA749892 | feces | Australia | A20 | Contig | 4.7 | 59 | 2022-01-25 |
| *A. veronii* | SAMN20667229 | JAIEXY01 | PRJNA749892 | feces | Australia | A21 | Contig | 4.5 | 59 | 2022-01-25 |
| *A. veronii* | SAMN20667230 | JAIEXZ01 | PRJNA749892 | feces | Australia | A26 | Contig | 4.5 | 59 | 2022-01-25 |
| *A. veronii* | SAMN20667231 | JAIEYA01 | PRJNA749892 | feces | Australia | A27 | Contig | 4.5 | 59 | 2022-01-25 |
| *A. veronii* | SAMN20667232 | JAIEYB01 | PRJNA749892 | feces | Australia | A7 | Contig | 4.5 | 59 | 2022-01-25 |
| *A. veronii* | SAMN20667233 | JAIEYC01 | PRJNA749892 | feces | Australia | A8 | Contig | 4.4 | 59 | 2022-01-25 |
| *A. veronii* | SAMN20667234 | JAIEYD01 | PRJNA749892 | feces | Australia | A9 | Contig | 4.5 | 59 | 2022-01-25 |
| *A. veronii* | SAMN20667235 | JAIEYE01 | PRJNA749892 | feces | Australia | A20-10 | Contig | 4.5 | 59 | 2022-01-25 |
| *A. veronii* | SAMN20667236 | JAIEYF01 | PRJNA749892 | feces | Australia | A20-12 | Contig | 4.5 | 59 | 2022-01-25 |
| *A. veronii* | SAMN20667237 | JAIEYG01 | PRJNA749892 | feces | Australia | A20-14 | Contig | 4.6 | 59 | 2022-01-25 |
| *A. veronii* | SAMN20667238 | JAIEYH01 | PRJNA749892 | feces | Australia | A20-17 | Contig | 4.5 | 59 | 2022-01-25 |
| *A. veronii* | SAMN20667239 | JAIEYI01 | PRJNA749892 | feces | Australia | A20-5 | Contig | 4.5 | 59 | 2022-01-25 |
| *A. veronii* | SAMN20667241 | JAIEYK01 | PRJNA749892 | feces | Australia | A21-10 | Contig | 4.4 | 59 | 2022-01-25 |
| *A. veronii* | SAMN20667242 | JAIEYL01 | PRJNA749892 | feces | Australia | A21-11 | Contig | 4.4 | 59 | 2022-01-25 |
| *A. veronii* | SAMN20667243 | JAIEYM01 | PRJNA749892 | feces | Australia | A21-13 | Contig | 4.4 | 59 | 2022-01-25 |
| *A. veronii* | SAMN20667244 | JAIEYN01 | PRJNA749892 | feces | Australia | A21-14 | Contig | 4.6 | 58 | 2022-01-25 |
| *A. veronii* | SAMN20667245 | JAIEYO01 | PRJNA749892 | feces | Australia | A21-15 | Contig | 4.5 | 59 | 2022-01-25 |
| *A. veronii* | SAMN20667246 | JAIEYP01 | PRJNA749892 | feces | Australia | A21-16 | Contig | 4.4 | 59 | 2022-01-25 |
| *A. veronii* | SAMN20667247 | JAIEYQ01 | PRJNA749892 | feces | Australia | A21-19 | Contig | 4.7 | 59 | 2022-01-25 |
| *A. veronii* | SAMN20667248 | JAIEYR01 | PRJNA749892 | feces | Australia | A21-4 | Contig | 4.6 | 59 | 2022-01-25 |
| *A. veronii* | SAMN20667249 | JAIEYS01 | PRJNA749892 | feces | Australia | A21-5 | Contig | 4.6 | 59 | 2022-01-25 |
| *A. veronii* | SAMN20667250 | JAIEYT01 | PRJNA749892 | feces | Australia | A21-6 | Contig | 4.5 | 59 | 2022-01-25 |
| *A. veronii* | SAMN20667251 | JAIEYU01 | PRJNA749892 | feces | Australia | A21-8 | Contig | 4.5 | 59 | 2022-01-25 |
| *A. veronii* | SAMN21168052 | JAJDSR01 | PRJNA759416 | bile | China:Ningbo | 71474 | Contig | 4.6 | 59 | 2021-10-26 |
| *A. veronii* | SAMN03455937 | LRBO01 | PRJNA279618 |  | USA | AVNIH2 | Contig | 4.5 | 59 | 2016-04-28 |
| *A. veronii* | SAMN04914378 | LXJN01 | PRJNA320014 | feces | India: Vellore | VBF557 | Contig | 4.7 | 58 | 2016-07-28 |
| *A. veronii* | SAMN05860752 | MRZR01 | PRJNA345311 | sputum | USA: Michigan | CCM 4359 | Contig | 4.5 | 59 | 2016-12-19 |
| *A. veronii* | SAMN10289379 | RHDQ01 | PRJNA498295 | feces | Brazil | 312M | Contig | 4.6 | 59 | 2018-12-03 |
| *A. veronii* | SAMN02463948 | AGWT01 | PRJNA71513 |  | not determined | AER39 | Scaffold | 4.4 | 59 | 2012-09-17 |
| *A. veronii* | SAMN02463949 | AGWU01 | PRJNA71515 |  | not determined | AMC34 | Scaffold | 4.6 | 58 | 2012-09-17 |
| *A. veronii* | SAMN02463950 | AGWV01 | PRJNA71517 |  | not determined | AER397 | Scaffold | 4.5 | 59 | 2012-09-17 |
| *A. veronii* | SAMN02463951 | AGWW01 | PRJNA71519 |  | not determined | AMC35 | Scaffold | 4.6 | 59 | 2012-09-17 |
| *A. veronii* | SAMN07312760 | NKWR01 | PRJNA391781 | blood | not available | BVH47 | Scaffold | 4.6 | 59 | 2019-06-11 |
| *A. veronii* | SAMN07312759 | NKWS01 | PRJNA391781 | blood | not available | BVH46 | Scaffold | 4.5 | 59 | 2019-06-11 |
| *A. veronii* | SAMN07312757 | NKWU01 | PRJNA391781 | blood | not available | BVH37 | Scaffold | 4.5 | 59 | 2019-06-11 |
| *A. veronii* | SAMN07312747 | NKXD01 | PRJNA391781 | Forehead abscess | not available | AK247 | Scaffold | 4.6 | 59 | 2019-06-11 |
| *A. veronii* | SAMN07312743 | NKXH01 | PRJNA391781 | stool | not available | ADV102 | Scaffold | 4.5 | 59 | 2019-06-11 |
| *A. veronii* | SAMN07680228 | NXBY01 | PRJNA408193 | rectal swab | China | ZJ12-3 | Scaffold | 4.7 | 58 | 2018-06-26 |
| *A. veronii* | SAMN08383672 | PPTE01 | PRJNA431081 | stool sample | China: Hangzhou | 126-14 | Scaffold | 4.4 | 59 | 2018-01-26 |
