# Supplementary material for: Clinical Features, Genome Epidemiology, and Antimicrobial Resistance Profiles of Aeromonas spp. Causing Human Infections: A Multicenter Prospective Cohort Study
Source: Open Forum Infect Dis. 2023 Nov 16;10(12):ofad587. doi: 10.1093/ofid/ofad587 (PMC10753922; doi:10.1093/ofid/ofad587)
Supplement: ofad587_Supplementary_Data [file ofad587_supplementary_data.zip › Supp_Table_5.docx]

**Supplementary Table 5.** The concordance between genotypic and phenotypic resistance for β-lactams.

| Species  (Number of the isolates) | The isolates carrying resistance genes  (% [number]) | | Concordant results between genotype and phenotype^*1^  (% [number]) | | | | | |
| --- | --- | --- | --- | --- | --- | --- | --- | --- |
|  | *bla*_AmpC_ | *bla*_CphA_ | 3^rd^ Gen. Cephalosporin^*2^ | CTX | CAZ | Carbapenem^*3^ | IPM | MEM |
| *A. caviae*  (n=87) | 99% (86) ^a^ | - | 58% (50) | 42% (36) | 17% (15) |  | - | - |
| *A. hydrophila*  (n=25) | 96% (24) ^b^ | 92% (23) | 17% (4) | 17% (4) | 4% (1) | 65% (15) | 65% (15) | 9% (2) |
| *A. dhakensis*  (n=9) | 100% (9) ^c^ | 100% (9) | 78% (7) | 67% (4) | 44% (4) | 100% (9) | 100% (9) | 33% (3) |
| *A. veronii*  (n=20) | - | 95.0% (19) |  | - | - | 53% (10) | 53% (10) | 16% (3) |

Abbreviations: CTX, cefotaxime; CAZ, ceftazidime; ATM, aztreonam; IPM, imipenem; MEM, meropenem; 3^rd^ Gen. Cephalosporin, 3^rd^ generation cephalosporin. ^a^ *bla*_MOX;_ ^b^ *bla*_CepH/CepS;_ ^c^ *bla*_AQU_.

**^*^**^1^ The Percentage (number) of the isolates showing non-susceptible testing results among those carrying resistance genes.

^*2^ The isolates showing non-susceptible testing results to at least one third generation cephalosporin (cefotaxime, ceftazidime).

^*3^ The isolates showing non-susceptible testing results to at least one carbapenem (imipenem, meropenem).
