# Supplementary material for: Clinical Features, Genome Epidemiology, and Antimicrobial Resistance Profiles of Aeromonas spp. Causing Human Infections: A Multicenter Prospective Cohort Study
Source: Open Forum Infect Dis. 2023 Nov 16;10(12):ofad587. doi: 10.1093/ofid/ofad587 (PMC10753922; doi:10.1093/ofid/ofad587)
Supplement: ofad587_Supplementary_Data [file ofad587_supplementary_data.zip › Supp_Table_6.docx]

**Supplementary Table 6.** The prevalence of antimicrobial resistance genes in *Aeromonas* species.

|  | Total  (n=144) | *A. caviae*  (n=87) | *A. hydrophila*  (n=25) | *A. veronii*  (n=20) | *A. dhakensis*  (n=9) | *A. allosaccharophila*  (n=2) | *A. media*  (n=1) |
| --- | --- | --- | --- | --- | --- | --- | --- |
| *bla*_CphA_ | 37% (53) | 0% (0) | 92% (23) | 95% (19) | 100% (9) | 100% (2) | 0% (0) |
| *bla*_MOX_ | 60% (87) | 99% (86) | 0% (0) | 0% (0) | 0% (0) | 0% (0) | 100% (1) |
| *bla*_AQU_ | 6.3% (9) | 0% (0) | 0% (0) | 0% (0) | 100% (9) | 0% (0) | 0% (0) |
| *bla*_CepH_ | 7.6% (11) | 0% (0) | 44% (11) | 0% (0) | 0% (0) | 0% (0) | 0% (0) |
| *bla*_CepS_ | 9.0% (13) | 0% (0) | 52 % (13) | 0% (0) | 0% (0) | 0% (0) | 0% (0) |
| *bla*_FOX-14_ | 1.4% (2) | 0% (0) | 0% (0) | 0% (0) | 0% (0) | 100% (2) | 0% (0) |
| *bla*_OXA-2-like_ | 0.7% (1) | 1.1% (1) | 0% (0) | 0% (0) | 0% (0) | 0% (0) | 0% (0) |
| *bla*_OXA-2_ | 0.7% (1) | 1.1% (1) | 0% (0) | 0% (0) | 0% (0) | 0% (0) | 0% (0) |
| *bla*_OXA-415_ | 0.7% (1) | 1.1% (1) | 0% (0) | 0% (0) | 0% (0) | 0% (0) | 0% (0) |
| *bla*_OXA-504-like_ | 60% (87) | 100% (87) | 0% (0) | 0% (0) | 0% (0) | 0% (0) | 0% (0) |
| *bla*_OXA-504_ | 49% (70) | 81% (70) | 0% (0) | 0% (0) | 0% (0) | 0% (0) | 0% (0) |
| *bla*_OXA-780_ | 9.0% (13) | 15% (13) | 0% (0) | 0% (0) | 0% (0) | 0% (0) | 0% (0) |
| *bla*_OXA-1037_ | 2.8% (4) | 4.6% (4) | 0% (0) | 0% (0) | 0% (0) | 0% (0) | 0% (0) |
| *bla*_OXA-12-like_ | 39% (56) | 0% (0) | 100% (25) | 100% (20) | 100% (9) | 100% (2) | 0% (0) |
| *bla*_OXA-950_ | 11 % (16) | 0% (0) | 56% (14) | 0% (0) | 22% (2) | 0% (0) | 0% (0) |
| *bla*_OXA-951_ | 3.5% (5) | 0% (0) | 4.0% (1) | 0% (0) | 44% (4) | 0% (0) | 0% (0) |
| *bla*_OXA-726_ | 6.9% (10) | 0% (0) | 28% (7) | 0% (0) | 33% (3) | 0% (0) | 0% (0) |
| *bla*_AmpH/OXA-724_ | 2.1% (3) | 0% (0) | 12% (3) | 0% (0) | 0% (0) | 0% (0) | 0% (0) |
| *bla*_OXA-912_ | 13% (19) | 0% (0) | 0% (0) | 95% (19) | 0% (0) | 0% (0) | 0% (0) |
| *bla*_AmpS/OXA-725_ | 0.7% (1) | 0% (0) | 0% (0) | 5.0% (1) | 0% (0) | 0% (0) | 0% (0) |
| *bla*_OXA-958_ | 0.7% (1) | 0% (0) | 0% (0) | 0% (0) | 0% (0) | 50% (1) | 0% (0) |
| *bla*_OXA-959_ | 0.7% (1) | 0% (0) | 0% (0) | 0% (0) | 0% (0) | 50% (1) | 0% (0) |
| *bla*_OXA-917_ | 0.7% (1) | 0% (0) | 0% (0) | 0% (0) | 0% (0) | 0% (0) | 100% (1) |
| *mcr*-3-like | 28% (40) | 3.4% (3) | 100% (25) | 15% (3) | 100% (9) | 0% (0) | 0% (0) |
| *mcr*-7-like | 13% (18) | 0% (0) | 0% (0) | 80% (16) | 0% (0) | 100% (2) | 0% (0) |
| *aadA1* | 2.8% (4) | 4.6% (4) | 0% (0) | 0% (0) | 0% (0) | 0% (0) | 0% (0) |
| *aadA2* | 2.1% (3) | 2.3% (2) | 0% (0) | 0% (0) | 11% (1) | 0% (0) | 0% (0) |
| *aph(3'')-Ib* | 0.7% (1) | 1.1% (1) | 0% (0) | 0% (0) | 0% (0) | 0% (0) | 0% (0) |
| *aph(3')-Ia* | 1.4% (2) | 1.1% (1) | 0% (0) | 0% (0) | 11% (1) | 0% (0) | 0% (0) |
| *aph(6)-Id* | 0.7% (1) | 1.1% (1) | 0% (0) | 0% (0) | 0% (0) | 0% (0) | 0% (0) |
| *dfrA12* | 1.4% (2) | 1.1% (1) | 0% (0) | 0% (0) | 11% (1) | 0% (0) | 0% (0) |
| *dfrA15* | 0.7% (1) | 1.1% (1) | 0% (0) | 0% (0) | 0% (0) | 0% (0) | 0% (0) |
| *sul1* | 4.2% (6) | 5.7% (5) | 0% (0) | 0% (0) | 11.1% (1) | 0% (0) | 0% (0) |
| *sul2* | 0.7% (1) | 1.1% (1) | 0% (0) | 0% (0) | 0% (0) | 0% (0) | 0% (0) |
| *qnrS2* | 0.7% (1) | 0% (0) | 4.0% (1) | 0% (0) | 0% (0) | 0% (0) | 0% (0) |
| *tet*(31) | 0.7% (1) | 1.1% (1) | 0% (0) | 0% (0) | 0% (0) | 0% (0) | 0% (0) |
| *tet*(A) | 0.7% (1) | 0% (0) | 0% (0) | 0% (0) | 11% (1) | 0% (0) | 0% (0) |
| *tet*(E) | 12% (17) | 12% (10) | 8.0% (2) | 15% (3) | 22% (2) | 0% (0) | 0% (0) |
| *mph*(A) | 2.8% (4) | 2.3% (2) | 4.0% (1) | 0% (0) | 11% (1) | 0% (0) | 0% (0) |
| *oqxB9* | 2.1% (3) | 3.4% (3) | 0% (0) | 0% (0) | 0% (0) | 0% (0) | 0% (0) |
| *cmlA4* | 0.7% (1) | 1.1% (1) | 0% (0) | 0% (0) | 0% (0) | 0% (0) | 0% (0) |
| *floR* | 0.7% (1) | 1.1% (1) | 0% (0) | 0% (0) | 0% (0) | 0% (0) | 0% (0) |
| *qacEΔ1* | 4.2% (6) | 5.7% (5) | 0% (0) | 0% (0) | 11% (1) | 0% (0) | 0% (0) |
| *catA* | 0.7% (1) | 1.1% (1) | 0% (0) | 0% (0) | 0% (0) | 0% (0) | 0% (0) |
| *catA2* | 0.7% (1) | 0% (0) | 0% (0) | 0% (0) | 11% (1) | 0% (0) | 0% (0) |

Data are percentage (number) of the isolates.
