# Supplementary material for: Clinical Features, Genome Epidemiology, and Antimicrobial Resistance Profiles of Aeromonas spp. Causing Human Infections: A Multicenter Prospective Cohort Study
Source: Open Forum Infect Dis. 2023 Nov 16;10(12):ofad587. doi: 10.1093/ofid/ofad587 (PMC10753922; doi:10.1093/ofid/ofad587)
Supplement: ofad587_Supplementary_Data [file ofad587_supplementary_data.zip › Supp_Table_7.docx]

**Supplementary Table 7.** Data availability of sequences reported in this study

| Isolates | Species | ST | BioProject | BioSample | Accession No. |
| --- | --- | --- | --- | --- | --- |
| FUJ80275 | *A. hydrophila* | 2018 | PRJNA896347 | SAMN31542812 | JAPEHN000000000 |
| FUJ80475 | *A. hydrophila* | 2019 | PRJNA896347 | SAMN31542813 | JAPEHM000000000 |
| FUJ80476 | *A. caviae* | 2020 | PRJNA896347 | SAMN31542814 | JAPEHL000000000 |
| FUJ80481 | *A. caviae* | 2021 | PRJNA896347 | SAMN31542815 | JAPEHK000000000 |
| FUJ80482 | *A. veronii* | 2022 | PRJNA896347 | SAMN31542816 | JAPEHJ000000000 |
| FUJ80484 | *A. veronii* | 2023 | PRJNA896347 | SAMN31542817 | JAPEHI000000000 |
| FUJ80487 | *A. dhakensis* | 2024 | PRJNA896347 | SAMN31542818 | JAPEHH000000000 |
| FUJ80488 | *A. caviae* | 2025 | PRJNA896347 | SAMN31542819 | JAPEHG000000000 |
| FUJ80490 | *A. caviae* | 2026 | PRJNA896347 | SAMN31542820 | JAPEHF000000000 |
| FUJ80491 | *A. veronii* | 2027 | PRJNA896347 | SAMN31542821 | JAPEHE000000000 |
| FUJ01298 | *A. hydrophila* | 517 | PRJNA896347 | SAMN31542822 | JAPEHD000000000 |
| FUJ01299 | *A. caviae* | 2028 | PRJNA896347 | SAMN31542823 | JAPEHC000000000 |
| FUJ01300 | *A. caviae* | 2029 | PRJNA896347 | SAMN31542824 | JAPEHB000000000 |
| FUJ01302 | *A. caviae* | 409 | PRJNA896347 | SAMN31542825 | JAPEHA000000000 |
| FUJ01303 | *A. caviae* | 2030 | PRJNA896347 | SAMN31542826 | JAPEGZ000000000 |
| FUJ01304 | *A. veronii* | 2031 | PRJNA896347 | SAMN31542827 | JAPEGY000000000 |
| FUJ01625 | *A. dhakensis* | 2032 | PRJNA896347 | SAMN31542828 | JAPEGX000000000 |
| FUJ01278 | *A. veronii* | 1686 | PRJNA896347 | SAMN31542829 | JAPEGW000000000 |
| FUJ01279 | *A. hydrophila* | 630 | PRJNA896347 | SAMN31542830 | JAPEGV000000000 |
| FUJ01280 | *A. caviae* | 2033 | PRJNA896347 | SAMN31542831 | JAPEGU000000000 |
| FUJ01282 | *A. caviae* | 2034 | PRJNA896347 | SAMN31542832 | JAPEGT000000000 |
| FUJ01283 | *A. hydrophila* | 367 | PRJNA896347 | SAMN31542833 | JAPEGS000000000 |
| FUJ01284 | *A. caviae* | 2035 | PRJNA896347 | SAMN31542834 | JAPEGR000000000 |
| FUJ01285 | *A. caviae* | 313 | PRJNA896347 | SAMN31542835 | JAPEGQ000000000 |
| FUJ01286 | *A. caviae* | 2036 | PRJNA896347 | SAMN31542836 | JAPEGP000000000 |
| FUJ01288 | *A. caviae* | 2037 | PRJNA896347 | SAMN31542837 | JAPEGO000000000 |
| FUJ01289 | *A. caviae* | 2038 | PRJNA896347 | SAMN31542838 | JAPEGN000000000 |
| FUJ01290 | *A. caviae* | 1610 | PRJNA896347 | SAMN31542839 | JAPEGM000000000 |
| FUJ01293 | *A. hydrophila* | 2039 | PRJNA896347 | SAMN31542840 | JAPEGL000000000 |
| FUJ01294 | *A. caviae* | 2040 | PRJNA896347 | SAMN31542841 | JAPEGK000000000 |
| FUJ01295 | *A. caviae* | 1825 | PRJNA896347 | SAMN31542842 | JAPEGJ000000000 |
| FUJ01296 | *A. caviae* | 2041 | PRJNA896347 | SAMN31542843 | JAPEGI000000000 |
| FUJ01297 | *A. caviae* | 2033 | PRJNA896347 | SAMN31542844 | JAPEGH000000000 |
| FUJ01809 | *A. hydrophila* | 2042 | PRJNA896347 | SAMN31542845 | JAPEGG000000000 |
| FUJ01810 | *A. veronii* | 2043 | PRJNA896347 | SAMN31542846 | JAPEGF000000000 |
| FUJ01812 | *A. caviae* | 1825 | PRJNA896347 | SAMN31542847 | JAPEGE000000000 |
| FUJ01814 | *A. dhakensis* | 795 | PRJNA896347 | SAMN31542848 | JAPEGD000000000 |
| FUJ01815 | *A. caviae* | 2044 | PRJNA896347 | SAMN31542849 | JAPEGC000000000 |
| FUJ01816 | *A. caviae* | 2045 | PRJNA896347 | SAMN31542850 | JAPEGB000000000 |
| FUJ01818 | *A. media* | 2046 | PRJNA896347 | SAMN31542851 | JAPEGA000000000 |
| FUJ01819 | *A. allosaccharophila* | 2047 | PRJNA896347 | SAMN31542852 | JAPEFZ000000000 |
| FUJ01821 | *A. caviae* | 2048 | PRJNA896347 | SAMN31542853 | JAPEFY000000000 |
| FUJ01822 | *A. caviae* | 2049 | PRJNA896347 | SAMN31542854 | JAPEFX000000000 |
| FUJ01829 | *A. veronii* | 2050 | PRJNA896347 | SAMN31542855 | JAPEFW000000000 |
| FUJ01830 | *A. caviae* | 2051 | PRJNA896347 | SAMN31542856 | JAPEFV000000000 |
| FUJ01831 | *A. caviae* | 2052 | PRJNA896347 | SAMN31542857 | JAPEFU000000000 |
| FUJ01833 | *A. caviae* | 2053 | PRJNA896347 | SAMN31542858 | JAPEFT000000000 |
| FUJ01834 | *A. caviae* | 2054 | PRJNA896347 | SAMN31542859 | JAPEFS000000000 |
| FUJ01837 | *A. veronii* | 2055 | PRJNA896347 | SAMN31542860 | JAPEFR000000000 |
| FUJ01838 | *A. caviae* | 2056 | PRJNA896347 | SAMN31542861 | JAPEFQ000000000 |
| FUJ01839 | *A. caviae* | 2057 | PRJNA896347 | SAMN31542862 | JAPEFP000000000 |
| FUJ01840 | *A. dhakensis* | 340 | PRJNA896347 | SAMN31542863 | JAPEFO000000000 |
| FUJ01841 | *A. hydrophila* | 2058 | PRJNA896347 | SAMN31542864 | JAPEFN000000000 |
| FUJ01842 | *A. caviae* | 2059 | PRJNA896347 | SAMN31542865 | JAPEFM000000000 |
| FUJ01843 | *A. caviae* | 1328 | PRJNA896347 | SAMN31542866 | JAPEFL000000000 |
| FUJ01844 | *A. hydrophila* | 2060 | PRJNA896347 | SAMN31542867 | JAPEFK000000000 |
| FUJ01845 | *A. veronii* | 2061 | PRJNA896347 | SAMN31542868 | JAPEFJ000000000 |
| FUJ01846 | *A. caviae* | 2062 | PRJNA896347 | SAMN31542869 | JAPEFI000000000 |
| FUJ01847 | *A. caviae* | 2054 | PRJNA896347 | SAMN31542870 | JAPEFH000000000 |
| FUJ01848 | *A. caviae* | 2063 | PRJNA896347 | SAMN31542871 | JAPEFG000000000 |
| FUJ01868 | *A. veronii* | 2064 | PRJNA896347 | SAMN31542872 | JAPEFF000000000 |
| FUJ01869 | *A. hydrophila* | 2065 | PRJNA896347 | SAMN31542873 | JAPEFE000000000 |
| FUJ01870 | *A. caviae* | 2066 | PRJNA896347 | SAMN31542874 | JAPEFD000000000 |
| FUJ01871 | *A. caviae* | 2066 | PRJNA896347 | SAMN31542875 | JAPEFC000000000 |
| FUJ80493 | *A. hydrophila* | 2067 | PRJNA896347 | SAMN31542876 | JAPEFB000000000 |
| FUJ80889 | *A. dhakensis* | 2068 | PRJNA896347 | SAMN31542877 | JAPEFA000000000 |
| FUJ80891 | *A. caviae* | 1825 | PRJNA896347 | SAMN31542878 | JAPEEZ000000000 |
| FUJ80892 | *A. caviae* | 2069 | PRJNA896347 | SAMN31542879 | JAPEEY000000000 |
| FUJ80894 | *A. hydrophila* | 2070 | PRJNA896347 | SAMN31542880 | JAPEEX000000000 |
| FUJ80897 | *A. dhakensis* | 518 | PRJNA896347 | SAMN31542881 | JAPEEW000000000 |
| FUJ80898 | *A. dhakensis* | 557 | PRJNA896347 | SAMN31542882 | JAPEEV000000000 |
| FUJ80900 | *A. veronii* | 923 | PRJNA896347 | SAMN31542883 | JAPEEU000000000 |
| FUJ01256 | *A. caviae* | 2071 | PRJNA896347 | SAMN31542884 | JAPEET000000000 |
| FUJ01259 | *A. caviae* | 2072 | PRJNA896347 | SAMN31542885 | JAPEES000000000 |
| FUJ01260 | *A. hydrophila* | 2073 | PRJNA896347 | SAMN31542886 | JAPEER000000000 |
| FUJ01261 | *A. caviae* | 2074 | PRJNA896347 | SAMN31542887 | JAPEEQ000000000 |
| FUJ01265 | *A. hydrophila* | 2075 | PRJNA896347 | SAMN31542888 | JAPEEP000000000 |
| FUJ01266 | *A. caviae* | 2076 | PRJNA896347 | SAMN31542889 | JAPEEO000000000 |
| FUJ01267 | *A. caviae* | 1968 | PRJNA896347 | SAMN31542890 | JAPEEN000000000 |
| FUJ01268 | *A. hydrophila* | 2077 | PRJNA896347 | SAMN31542891 | JAPEEM000000000 |
| FUJ01270 | *A. caviae* | 2078 | PRJNA896347 | SAMN31542892 | JAPEEL000000000 |
| FUJ01275 | *A. caviae* | 2079 | PRJNA896347 | SAMN31542893 | JAPEEK000000000 |
| FUJ01736 | *A. caviae* | 2080 | PRJNA896347 | SAMN31542894 | JAPEEJ000000000 |
| FUJ01737 | *A. caviae* | 2081 | PRJNA896347 | SAMN31542895 | JAPEEI000000000 |
| FUJ01739 | *A. caviae* | 2082 | PRJNA896347 | SAMN31542896 | JAPEEH000000000 |
| FUJ01742 | *A. hydrophila* | 2083 | PRJNA896347 | SAMN31542897 | JAPEEG000000000 |
| FUJ01743 | *A. caviae* | 2084 | PRJNA896347 | SAMN31542898 | JAPEEF000000000 |
| FUJ01746 | *A. caviae* | 2085 | PRJNA896347 | SAMN31542899 | JAPEEE000000000 |
| FUJ01747 | *A. allosaccharophila* | 2086 | PRJNA896347 | SAMN31542900 | JAPEED000000000 |
| FUJ01748 | *A. caviae* | 2087 | PRJNA896347 | SAMN31542901 | JAPEEC000000000 |
| FUJ01749 | *A. caviae* | 313 | PRJNA896347 | SAMN31542902 | JAPEEB000000000 |
| FUJ01756 | *A. caviae* | 2088 | PRJNA896347 | SAMN31542903 | JAPEEA000000000 |
| FUJ01757 | *A. caviae* | 1554 | PRJNA896347 | SAMN31542904 | JAPEDZ000000000 |
| FUJ01761 | *A. caviae* | 2089 | PRJNA896347 | SAMN31542905 | JAPEDY000000000 |
| FUJ01765 | *A. caviae* | 368 | PRJNA896347 | SAMN31542906 | JAPEDX000000000 |
| FUJ01766 | *A. veronii* | 2090 | PRJNA896347 | SAMN31542907 | JAPEDW000000000 |
| FUJ81138 | *A. veronii* | 2091 | PRJNA896347 | SAMN31542908 | JAPEDV000000000 |
| FUJ81139 | *A. caviae* | 2092 | PRJNA896347 | SAMN31542909 | JAPEDU000000000 |
| FUJ81140 | *A. veronii* | 2064 | PRJNA896347 | SAMN31542910 | JAPEDT000000000 |
| FUJ81141 | *A. veronii* | 2093 | PRJNA896347 | SAMN31542911 | JAPEDS000000000 |
| FUJ81143 | *A. veronii* | 2094 | PRJNA896347 | SAMN31542912 | JAPEDR000000000 |
| FUJ81167 | *A. hydrophila* | 2095 | PRJNA896347 | SAMN31542913 | JAPEDQ000000000 |
| FUJ81169 | *A. dhakensis* | 2096 | PRJNA896347 | SAMN31542914 | JAPEDP000000000 |
| FUJ81171 | *A. veronii* | 2097 | PRJNA896347 | SAMN31542915 | JAPEDO000000000 |
| FUJ81172 | *A. caviae* | 2098 | PRJNA896347 | SAMN31542916 | JAPEDN000000000 |
| FUJ81174 | *A. caviae* | 368 | PRJNA896347 | SAMN31542917 | JAPEDM000000000 |
| FUJ81177 | *A. caviae* | 2099 | PRJNA896347 | SAMN31542918 | JAPEDL000000000 |
| FUJ81179 | *A. caviae* | 409 | PRJNA896347 | SAMN31542919 | JAPEDK000000000 |
| FUJ81180 | *A. caviae* | 2100 | PRJNA896347 | SAMN31542920 | JAPEDJ000000000 |
| FUJ81181 | *A. caviae* | 1473 | PRJNA896347 | SAMN31542921 | JAPEDI000000000 |
| FUJ81182 | *A. caviae* | 2101 | PRJNA896347 | SAMN31542922 | JAPEDH000000000 |
| FUJ01717 | *A. caviae* | 2102 | PRJNA896347 | SAMN31542923 | JAPEDG000000000 |
| FUJ01718 | *A. caviae* | 2103 | PRJNA896347 | SAMN31542924 | JAPEDF000000000 |
| FUJ01719 | *A. caviae* | 1481 | PRJNA896347 | SAMN31542925 | JAPEDE000000000 |
| FUJ01720 | *A. caviae* | 2104 | PRJNA896347 | SAMN31542926 | JAPEDD000000000 |
| FUJ01721 | *A. veronii* | 2105 | PRJNA896347 | SAMN31542927 | JAPEDC000000000 |
| FUJ01723 | *A. hydrophila* | 2106 | PRJNA896347 | SAMN31542928 | JAPEDB000000000 |
| FUJ01224 | *A. caviae* | 2107 | PRJNA896347 | SAMN31542929 | JAPEDA000000000 |
| FUJ01227 | *A. dhakensis* | 2108 | PRJNA896347 | SAMN31542930 | JAPECZ000000000 |
| FUJ01228 | *A. caviae* | 2109 | PRJNA896347 | SAMN31542931 | JAPECY000000000 |
| FUJ01229 | *A. hydrophila* | 721 | PRJNA896347 | SAMN31542932 | JAPECX000000000 |
| FUJ01230 | *A. caviae* | 1825 | PRJNA896347 | SAMN31542933 | JAPECW000000000 |
| FUJ01232 | *A. hydrophila* | 2110 | PRJNA896347 | SAMN31542934 | JAPECV000000000 |
| FUJ01235 | *A. hydrophila* | 2111 | PRJNA896347 | SAMN31542935 | JAPECU000000000 |
| FUJ01237 | *A. caviae* | 2112 | PRJNA896347 | SAMN31542936 | JAPECT000000000 |
| FUJ01238 | *A. caviae* | 2112 | PRJNA896347 | SAMN31542937 | JAPECS000000000 |
| FUJ01239 | *A. veronii* | 2113 | PRJNA896347 | SAMN31542938 | JAPECR000000000 |
| FUJ01240 | *A. caviae* | 2114 | PRJNA896347 | SAMN31542939 | JAPECQ000000000 |
| FUJ01241 | *A. caviae* | 2115 | PRJNA896347 | SAMN31542940 | JAPECP000000000 |
| FUJ01243 | *A. caviae* | 2116 | PRJNA896347 | SAMN31542941 | JAPECO000000000 |
| FUJ01892 | *A. caviae* | 2117 | PRJNA896347 | SAMN31542942 | JAPECN000000000 |
| FUJ01895 | *A. caviae* | 2114 | PRJNA896347 | SAMN31542943 | JAPECM000000000 |
| FUJ01896 | *A. hydrophila* | 2118 | PRJNA896347 | SAMN31542944 | JAPECL000000000 |
| FUJ01898 | *A. caviae* | 2119 | PRJNA896347 | SAMN31542945 | JAPECK000000000 |
| FUJ01899 | *A. caviae* | 2120 | PRJNA896347 | SAMN31542946 | JAPECJ000000000 |
| FUJ01902 | *A. caviae* | 2121 | PRJNA896347 | SAMN31542947 | JAPECI000000000 |
| FUJ01903 | *A. hydrophila* | 2122 | PRJNA896347 | SAMN31542948 | JAPECH000000000 |
| FUJ01904 | *A. hydrophila* | 2123 | PRJNA896347 | SAMN31542949 | JAPECG000000000 |
| FUJ01905 | *A. caviae* | 2124 | PRJNA896347 | SAMN31542950 | JAPECF000000000 |
| FUJ01907 | *A. hydrophila* | 2125 | PRJNA896347 | SAMN31542951 | JAPECE000000000 |
| FUJ01909 | *A. veronii* | 2126 | PRJNA896347 | SAMN31542952 | JAPECD000000000 |
| FUJ01911 | *A. caviae* | 2127 | PRJNA896347 | SAMN31542953 | JAPECC000000000 |
| FUJ01912 | *A. caviae* | 2128 | PRJNA896347 | SAMN31542954 | JAPECB000000000 |
| FUJ01916 | *A. caviae* | 2129 | PRJNA896347 | SAMN31542955 | JAPECA000000000 |
