# Supplementary material for: Clinical Features, Genome Epidemiology, and Antimicrobial Resistance Profiles of Aeromonas spp. Causing Human Infections: A Multicenter Prospective Cohort Study
Source: Open Forum Infect Dis. 2023 Nov 16;10(12):ofad587. doi: 10.1093/ofid/ofad587 (PMC10753922; doi:10.1093/ofid/ofad587)
Supplement: ofad587_Supplementary_Data [file ofad587_supplementary_data.zip › Supplementary Appendix_1101.docx]

**Supplementary Appendix**

**Supplementary Figure.** Core genome phylogeny of the genomes obtained in this study combined with publicly available *Aeromonas* genomes of human origin, for 4 major *Aeromonas* species (*A. caviae*, *A. hydrophila*, *A. veronii*, and *A. dhakensis*). An unrooted maximum-likelihood phylogenetic tree was constructed based on SNPs analysis, with a type-strain genome of the corresponding species used as the reference. The phylogeny was annotated with geographic regions where publicly available genomes were collected.

**Supplementary Table 1.**  Reference genomes representing 30 species of the genus A*eromonas.*

**Supplementary Table 2.** Pathogenic bacteria co-isolated with *Aeromonas* spp. from clinical specimens, stratified into those with and without hepatobiliary infections.

**Supplementary Table 3.** *Aeromonas* species isolated from each infection site.

**Supplementary Table 4.**  *Aeromonas* genomes registered in GenBank, isolated from human sources.

**Supplementary Table 5.**  The concordance between genotypic and phenotypic resistance for β-lactams.

**Supplementary Table 6.** The prevalence of antimicrobial resistance genes in *Aeromonas* species.

**Supplementary Table 7.** Data availability of sequences reported in this study.
