# Supplementary material for: Clinical Features, Genome Epidemiology, and Antimicrobial Resistance Profiles of Aeromonas spp. Causing Human Infections: A Multicenter Prospective Cohort Study
Source: Open Forum Infect Dis. 2023 Nov 16;10(12):ofad587. doi: 10.1093/ofid/ofad587 (PMC10753922; doi:10.1093/ofid/ofad587)
Supplement: ofad587_Supplementary_Data [file ofad587_supplementary_data.zip › Supplementary Materials_0807.docx]

**Supplementary Materials**

1. Clinical definitions
2. Statistical analysis
3. Whole genome sequencing and Bioinformatics
4. **Clinical Definitions**
5. ***Definition of infections***

A patient with a culture positive for *Aeromonas* spp. from a blood or any other sterile source was deemed to have an infection. For a patient with a culture positive from a non-sterile site, an infection was defined using established standard criteria, as detailed below. Otherwise, a positive culture was deemed as colonization.

For patients with a positive culture from respiratory, urine, surgical wound, skin-soft tissue, intra-abdominal, or stool specimens, the Centers for Disease Control and Prevention (CDC) / National Healthcare Safety Network (NHSN) criteria for pneumonia (clinically defined pneumonia), urinary tract infection, surgical site infection, skin and soft tissue infection, intraabdominal infection, or gastroenteritis were applied, respectively [1,2]. For those with a positive culture from specimens obtained from the biliary tract (e.g., bile), the definite diagnostic criteria for acute cholangitis or cholecystitis outlined in the Tokyo guideline 2018 were applied [3–5].

Bacteremia was classified into primary and secondary bacteremia. Secondary bacteremia was defined when a primary site of infection was identified according to the above-mentioned site-specific infection criteria and/or by the isolation of an identical *Aeromonas* strain from the site-specific culture [2]. For patients with secondary bacteremia, the original infection site was deemed their infection site. For those with primary bacteremia (i.e., bacteremia with an unknown source), primary bacteremia itself was considered as an infection site.

1. ***Clinical data collection and definition***

Immunocompromised status includes one or more of the following at the time of diagnosis [6,7]: neutropenia (<0.5 × 10^9^ neutrophils/L), corticosteroid use (at least ≥0.3 mg/kg corticosteroids for ≥3 weeks) in the past 60 days, immunosuppressant use (e.g., calcineurin inhibitors, anti-tumor necrosis factor inhibitors, lymphocyte-specific monoclonal antibodies, immunosuppressive nucleoside analogs) in the past 90 days, cytotoxic chemotherapy in the past 30 days, history of allogeneic stem cell transplant or solid organ transplant, or HIV infection with CD4-positive lymphocytes < 200/mL or an AIDS-defining condition.

Infection types were classified into hospital-acquired, healthcare-associated, and community-acquired [8,9]. Briefly, hospital-acquired infection was defined as an infection documented in patients who had been hospitalized for 48 hours or longer [8]. Healthcare- associated infection was defined as infection that was present at the time of or developed within 48 hours of hospital admission in patients with a recent history of medical care (e.g., hospitalization in an acute care hospital for 2 or more days within 90 days, receipt of intravenous chemotherapy within 30 days, or residence in a nursing home or long-term-care facility) [9]. Community-acquired infection was defined as an infection that was present at the time of or developed within the 48 hours of hospital admission in patients who did not fulfill the criteria for a healthcare-associated infection.

Hepatobiliary complications were defined as anatomical or functional factors that compromise the hepatobiliary tract, including obstruction or stricture (e.g., stone in the bile duct, cholangiocarcinoma), the presence of artificial materials

(e.g., biliary tube/stent) and history of surgeries (e.g., bile duct resection and reconstruction, pancreatoduodenectomy) [3,4]. In those with hepatobiliary infections, the length of time from diagnosis to initiation of effective antibiotics and implementation of source control measures was evaluated. Antimicrobial therapy was considered “appropriate” when the regimen contained antibiotics that were active *in vitro* against the *Aeromonas* strain isolated.

1. **Statistical Analysis**

Differences in the incidence of *Aeromonas* infections between months with average monthly temperatures above and below 20℃ were evaluated using ordinal logistic regression models. The number of *Aeromonas* infections was calculated per facility per month as the average for 2020-2022 and treated as ordinal categorical data since the scale was not large enough to be considered a continuous variable. The monthly average temperatures were determined using data from Japan Meteorological Agency (https://www.data.jma.go.jp/). For the cumulative number of infections per calendar month during the study period at each facility, the variance of the regression coefficients was estimated using Huber-White's robust variance with facilities as clusters.

We evaluated differences in species distribution among infection sites using frequency and Fisher's exact test, and differences in antimicrobial susceptibility testing results between species were assessed using the Kruskal-Wallis test. All p-values were two-sided, and p-values <0.05 were considered statistically significant. The statistical analysis was performed using STATA 15.1 and R version 4.3.1 (http://www.r-project.org).

1. **Whole genome sequencing and Bioinformatics**
2. ***Whole genome sequencing***

Genomic DNA was extracted from the isolates using the DNeasy Blood and Tissue Kit

(Qiagen, Tokyo, Japan) per the manufacturer's instructions. Whole genome sequencing was performed with the NextSeq 2000 platform (Illumina, Inc., San Diego, CA, USA) using 2 ×150-bp paired-end reads, followed by *de novo* genome assembly with SPAdes v3.13.1 [10].

1. ***Phylogenetic analysis***

A single-nucleotide polymorphism (SNP) was extracted from the core-genome alignment using Snippy v4.6.0 (https://github.com/tseemann/snippy.git), with the *A. hydrophila* ATCC7966^T^ genome (GenBank accession no. CP000462.1) used as the reference, unless otherwise indicated. A variant call required a minimum base quality of 13 and read coverage of 10, with an allele frequency of 0.9% at the locus. The full SNP alignments were fed to RAxML (Randomized Axelerated Maximum Likelihood, v8.2.11) to build a maximum likelihood phylogenetic tree with 100 bootstrap iterations. A phylogenetic tree was visualized and annotated utilizing iTOL v6 (https://itol.embl.de).

1. ***Species Identification***

First, genome sequencing data of type strains of 30 *Aeromonas* species that have been validly published under the List of Prokaryotic names with Standing in Nomenclature (LPSN) ([https://www.bacterio.net/](about:blank)) [11] were downloaded from the National Institutes of Health (NIH) genetic sequence database (GenBank) on June 20, 2022. Among them, type strain genomes were selected for each species and treated as reference genomes. For *A.* *rivipollensis,* the genome of a representative strain was used since a type strain genome was not available. The reference genome set representing 30 species is listed in Supplementary Table 1. Second, a core-genome SNPs-based maximum likelihood phylogenetic tree was built using the genome sequences obtained in this study (i.e., query genomes) combined with 30 reference genomes. Lastly, OrthoANI values were calculated between the query genomes and the reference genomes that clustered together in a single clade in the phylogenetic tree, with an OrthoANI cut-off value of 95% used for species delineation [12–14].

1. ***Multilocus sequencing typing***

*In silico* multilocus sequencing typing (MLST) was performed using MLST v2.0.9 which are available from the Center for Genomic Epidemiology [(http://genomicepidemiology.org/](file:///C:\Users\ajian\Dropbox\FUJITA%20research\Aeromonas%20spp\自院および他院からの菌株リスト\(http:\genomicepidemiology.org\)), utilizing internal blast. Novel allele sequences and MLST profiles were submitted to PubMLST ([https://pubmlst.org/organisms/aeromonas-spp](file:///C:\Users\ajian\Dropbox\FUJITA%20research\Aeromonas%20spp\自院および他院からの菌株リスト\ https:\pubmlst.org\organisms\aeromonas-spp)) [15] for assignment.

1. ***Phylogenetic analysis to illustrate the genetic relatedness to publicly available genomes isolated from human sources***

Genome sequence data of 4 *Aeromona*s spp. (*A. caviae*, *A. hydrophila*, *A. veronii*, and *A. dhakensis*) registered in GenBank as of November 7, 2022 and labelled to be isolated from “*Homo sapiens*” were downloaded. There were 50, 34, 67, and 27 genomes belonging to *A. caviae*, *A. hydrophila*, *A. veronii*, and *A. dhakensis*, respectively. These downloaded genomes were combined with genome sequences derived from study isolates and used to build a con-specific phylogenetic tree using the above-mentioned method. The reference genome (i.e., type strain genome, Supplementary Table 1) of corresponding species was used as the reference to call SNPs.

1. ***Antimicrobial resistance gene identification.***

Antimicrobial resistance (AMR) genes were searched using AMRFinderPlus (<https://www.ncbi.nlm.nih.gov/pathogens/antimicrobial-resistance/AMRFinder/>) [16] with initial parameters including a minimum identity of 70% over 50% of the coverage. Eventually, AMR genes with an identity higher than 85% and a coverage higher than 90% were called present, except for the genes encoding mobile colistin resistance (MCR). For *mcr* genes, where a wide variety of percentage identities were observed within the group [17], in accordance with the proposal by Partrige SR et al. [18], *mcr*-genes with coverage ≧90% were called as “*mcr*-like,” irrespective of their percentage identities. The location of *mcr*-like genes was examined by comparative genomic analysis between a contig containing *mcr*-like gene in study isolates and an annotated complete genome sequence of corresponding species (*A. hydrophila* [GenBank Acc No. CP050851], *A. dhakensis* [Acc No. CP084351], *A. veronii* [Acc No. CP044060]), using BLAST and Artemis comparison tool (https://www.sanger.ac.uk/tool/artemis/)

**References**

1. Horan TC, Andrus M, Dudeck MA. CDC/NHSN surveillance definition of health care–associated infection and criteria for specific types of infections in the acute care setting. American Journal of Infection Control **2008**; 36:309–332.

2. Centers for Disease Control and Prevention. National Healthcare Safety Network (NHSN) patient safety component manual 2021;(January):1–39. Atlanta: CDC

3. Kiriyama S, Kozaka K, Takada T, et al. Tokyo Guidelines 2018: diagnostic criteria and severity grading of acute cholangitis (with videos). J Hepatobiliary Pancreat Sci **2018**; 25:17–30.

4. Yokoe M, Hata J, Takada T, et al. Tokyo Guidelines 2018: diagnostic criteria and severity grading of acute cholecystitis (with videos). J Hepatobiliary Pancreat Sci **2018**; 25:41–54.

5. Miura F, Okamoto K, Takada T, et al. Tokyo Guidelines 2018: initial management of acute biliary infection and flowchart for acute cholangitis. J Hepatobiliary Pancreat Sci **2018**; 25:31–40.

6. Donnelly JP, Chen SC, Kauffman CA, et al. Revision and Update of the Consensus Definitions of Invasive Fungal Disease From the European Organization for Research and Treatment of Cancer and the Mycoses Study Group Education and Research Consortium. Clin Infect Dis **2019**; 71:1367–1376.

7. Harris PNA, Tambyah PA, Lye DC, et al. Effect of Piperacillin-Tazobactam vs Meropenem on 30-Day Mortality for Patients With E coli or Klebsiella pneumoniae Bloodstream Infection and Ceftriaxone Resistance: A Randomized Clinical Trial. JAMA **2018**; 320:984–994.

8. Garner JS, Jarvis WR, Emori TG, Horan TC, Hughes JM. CDC definitions for nosocomial infections, 1988. Am J Infect Control **1988**; 16:128–140.

9. Friedman ND, Kaye KS, Stout JE, et al. Health care-associated bloodstream infections in adults: a reason to change the accepted definition of community-acquired infections. Ann Intern Med **2002**; 137:791–797.

10. Bankevich A, Nurk S, Antipov D, et al. SPAdes: a new genome assembly algorithm and its applications to single-cell sequencing. J Comput Biol **2012**; 19:455–477.

11. Parte AC, Sardà Carbasse J, Meier-Kolthoff JP, Reimer LC, Göker M. List of Prokaryotic names with Standing in Nomenclature (LPSN) moves to the DSMZ. Int J Syst Evol Microbiol **2020**; 70:5607–5612.

12. Lee I, Kim YO, Park SC, Chun J. OrthoANI: An improved algorithm and software for calculating average nucleotide identity. International Journal of Systematic and Evolutionary Microbiology **2016**; 66:1100–1103.

13. Goris J, Konstantinidis KT, Klappenbach JA, Coenye T, Vandamme P, Tiedje JM. DNA-DNA hybridization values and their relationship to whole-genome sequence similarities. Int J Syst Evol Microbiol **2007**; 57:81–91.

14. Colston SM, Fullmer MS, Beka L, Lamy B, Peter Gogarten J, Graf J. Bioinformatic genome comparisons for taxonomic and phylogenetic assignments using aeromonas as a test case. mBio **2014**; 5:1–13.

15. Jolley KA, Bray JE, Maiden MCJ. Open-access bacterial population genomics: BIGSdb software, the PubMLST.org website and their applications. Wellcome Open Res **2018**; 3:124.

16. Feldgarden M, Brover V, Haft DH, et al. Validating the AMRFinder Tool and Resistance Gene Database by Using Antimicrobial Resistance Genotype-Phenotype Correlations in a Collection of Isolates. Antimicrob Agents Chemother **2019**; 63:e00483-19.

17. Hall RM, Schwarz S. Resistance gene naming and numbering: is it a new gene or not? J Antimicrob Chemother **2016**; 71:569–571.

18. Partridge SR, Di Pilato V, Doi Y, et al. Proposal for assignment of allele numbers for mobile colistin resistance (mcr) genes. Journal of Antimicrobial Chemotherapy **2018**; 73:2625–2630.
